# GPVI (Glycoprotein VI) Interaction With Fibrinogen Is Mediated by Avidity and the Fibrinogen αC-Region

**DOI:** 10.1161/ATVBAHA.120.315030

**Published:** 2021-01-21

**Authors:** Rui-Gang Xu, Julia S. Gauer, Stephen R. Baker, Alexandre Slater, Eleyna M. Martin, Helen R. McPherson, Cédric Duval, Iain W. Manfield, Arkadiusz M. Bonna, Steve P. Watson, Robert A.S. Ariëns

**Affiliations:** 1 Discovery and Translational Science Department, Institute of Cardiovascular and Metabolic Medicine (R.-G.X., J.S.G., S.R.B., H.R.M., C.D., R.A.S.A.); 2 School of Molecular and Cellular Biology, Faculty of Biological Sciences (I.W.M.), University of Leeds, United Kingdom.; 3 Department of Physics, Wake Forest University, Winston Salem, NC (S.R.B.).; 4 Institute of Cardiovascular Sciences, College of Medical and Dental Sciences, University of Birmingham, United Kingdom (A.S., E.M.M., S.P.W.).; 5 Department of Biochemistry, University of Cambridge, United Kingdom (A.M.B.).

**Keywords:** fibrin, fibrinogen, glycoprotein VI, platelet aggregation, thrombosis

## Abstract

**Objective:**

GPVI (glycoprotein VI) is a key molecular player in collagen-induced platelet signaling and aggregation. Recent evidence indicates that it also plays important role in platelet aggregation and thrombus growth through interaction with fibrin(ogen). However, there are discrepancies in the literature regarding whether the monomeric or dimeric form of GPVI binds to fibrinogen at high affinity. The mechanisms of interaction are also not clear, including which region of fibrinogen is responsible for GPVI binding. We aimed to gain further understanding of the mechanisms of interaction at molecular level and to identify the regions on fibrinogen important for GPVI binding.

**Approach and Results:**

Using multiple surface- and solution-based protein-protein interaction methods, we observe that dimeric GPVI binds to fibrinogen with much higher affinity and has a slower dissociation rate constant than the monomer due to avidity effects. Moreover, our data show that the highest affinity interaction of GPVI is with the αC-region of fibrinogen. We further show that GPVI interacts with immobilized fibrinogen and fibrin variants at a similar level, including a nonpolymerizing fibrin variant, suggesting that GPVI binding is independent of fibrin polymerization.

**Conclusions:**

Based on the above findings, we conclude that the higher affinity of dimeric GPVI over the monomer for fibrinogen interaction is achieved by avidity. The αC-region of fibrinogen appears essential for GPVI binding. We propose that fibrin polymerization into fibers during coagulation will cluster GPVI through its αC-region, leading to downstream signaling, further activation of platelets, and potentially stimulating clot growth.

## Abstract 2

Supplemental Digital Content is available in the text.

**Highlights**

- Fibrinogen interacts with monomeric and dimeric GPVI (glycoprotein VI) with distinct affinities and kinetic profiles. The higher affinity of dimeric GPVI for fibrinogen is due to increased avidity.
- The αC-region of fibrinogen is crucial for high-affinity GPVI binding and plays a more important role than other fibrinogen regions in the interaction.
- Fibrin polymerization is not required for GPVI binding, as similar binding profiles of GPVI monomer and dimer to various fibrinogen and fibrin variants were observed.

GPVI (glycoprotein VI), expressed by platelets and megakaryocytes, is widely accepted as an activation receptor for collagen, playing a critical role in platelet activation and thrombus initiation,^1,2^ as well as in prevention of inflammation-induced hemorrhage.^3^ The importance of GPVI in thrombus formation is emphasized by observations that absence of GPVI impairs experimental thrombosis in mouse models, including no occlusive thrombus formation observed in knockout mice following FeCl_3_ injury.^4–6^ Conversely, absence of GPVI in mice has minor effects on haemostasis, and GPVI-deficient patients present with a mild bleeding diathesis.^7–9^ These observations suggest that GPVI is a promising antithrombotic target with a minor impact on hemostasis.^10^ Upon vessel injury, GPVI interacts with exposed subendothelial collagen, resulting in platelet signaling, activation, and thrombus formation.^11^ The extracellular region of GPVI consists of 2 IgG-like domains (D1 and D2) and is closely related to Fcα**R** and natural killer receptors of the immunoglobulin superfamily.^12,13^

Fibrinogen is essential for platelet aggregation and blood coagulation. It contains 2 distal D-regions and a central E-region, connected by 2 α-helical coiled-coils. Each fibrinogen molecule has 2 αC-regions, composed by the C-terminal two-thirds of the Aα-chains^14^ (Figure I in the Data Supplement). It has been suggested that the αC-region folds back from the distal end and interacts with the E-region via its relatively compact C-terminal domain.^15,16^ Initiation of fibrin polymerization is triggered by thrombin, through knob-hole interactions between the D- and E-regions of adjacent molecules. Polymerized fibrin is strengthened by FXIIIa (factor XIIIa)-mediated intermolecular cross-linking of the αC- and D-regions. Early-stage fibrinogen degradation products by plasmin or trypsin include X-fragment (truncated αC-region)^16^ and Y-fragment (truncated αC-region and one of the 2 D-regions). Later-stage degradation products D- and E-fragments are released during prolonged proteolysis time. D-dimer is a final degradation product of polymerized fibrin that has been cross-linked by FXIIIa (Figure I in the Data Supplement).^17,18^

Recently, a role for GPVI in thrombus growth independent of its interaction with collagen has been reported, with several studies indicating that GPVI binds fibrin(ogen).^19–24^ Although one study showed binding of monomeric GPVI extracellular domain to immobilized fibrinogen^19^ and another detected low levels of dimeric GPVI (extracellular domain fused by IgG-Fc, also called GPVI-Fc or GPVI-Fc_2_) binding to fibrinogen,^22^ all of the other reports focused on GPVI binding to fibrin.^20,21,23^ The breakdown product of fibrin, D-dimer, has been shown to bind GPVI in 2 reports^22,23^ and to act as a competitive inhibitor in displacement studies.^19,23^ Collagen and CRP-XL (cross-linked collagen-related peptide) also displace D-dimer/D-fragment binding, suggesting that the binding site of collagen and fibrin(ogen) on GPVI may overlap.^22^ All previous interaction studies used immobilized fibrin(ogen) or its fragments. Whether fibrinogen and fibrin also interact with GPVI in solution is unclear as immobilized fibrin(ogen) may adopt a different configuration. Another unresolved question is whether other regions of fibrinogen interact with GPVI. Finally, there are discrepancies in the literature as to whether monomeric or dimeric GPVI (Figure II in the Data Supplement) shows higher affinity for fibrin(ogen).^25^

Here, we investigate the molecular interaction mechanisms of GPVI and fibrin(ogen), using a combination of 4 different protein-protein interaction methods, that is, ELISA, microscale thermophoresis (MST), surface plasmon resonance (SPR), and atomic force microscopy (AFM), to gain detailed understanding of the binding affinity and kinetics while also identifying the GPVI binding regions on the fibrinogen molecule. We also investigate the effects of fibrin polymerization on GPVI binding using a novel recombinant fibrinogen mutant that is unable to polymerize on conversion to fibrin by thrombin, called nonpolymerizing (previously also called double-Detroit in Duval et al^26^) fibrinogen. Our data show that both monomeric and dimeric GPVI bind the αC-region of fibrinogen and that their affinities and kinetic profiles are distinct from each other based on avidity. Binding is independent of fibrin polymerization as similar binding profiles were observed for recombinant wild-type, nonpolymerizing, and plasma-purified fibrin.

## Materials and Methods

The data that support the findings of this study are available from the corresponding author upon reasonable request.

### Enzyme-Linked Immunosorbent Assays

ELISAs were performed based on previously described methods,^23^ with slight modifications. In brief, 96-well Maxisorp Nunc-immuno plates were coated with 10 nmol/L BSA, purified fibrinogen, type 1 collagen fibrils or CRP-XL, or 100 nmol/L GPVI-Fc/GPVI monomer, in duplicates overnight at 4 °C. Immobilized fibrinogen was converted to fibrin by addition of 0.1 U/mL thrombin for 20 minutes at room temperature. Plates were washed to remove excess protein and blocked with 3% BSA-PBS for 1 hour at room temperature. After washing, plates were incubated with purified GPVI constructs at 100 nmol/L, or BSA control, for 1 hour. For concentration-dependent experiments, 0 to 400 nmol/L GPVI constructs were used, and 100 nmol/L CRP-XL was preincubated with matching concentrations of GPVI constructs for 15 minutes in competition assays. In addition, for GPVI displacement experiments 0 to 100 nmol/L of either GPVI construct was preincubated with 100 nmol/L of the alternative construct for 15 minutes before addition to the plate. After incubation with GPVI constructs, plates were washed and incubated for 1 hour at room temperature with either 1:10 000 goat anti-human IgG-Fc HRP (horseradish peroxidase)-conjugated antibody to detect binding of dimeric GPVI-Fc or 1:10 000 rabbit anti–6-His HRP-conjugated antibody to detect monomeric His-tagged GPVI. After washing, the binding of GPVI to immobilized substrate was visualized using 3,3’,5,5’-tetramethylbenzidine. The reaction was stopped by addition of 1 mol/L H_2_SO_4_ and the optical density was read at 450 nm. All binding values obtained were normalized to their corresponding BSA controls to account for background binding levels. Results were expressed as an average of 3 repeats ±SEM.

### Microscale Thermophoresis

Each of the 16 binding reactions was prepared by mixing 10 μL of 50 nmol/L fluorescein isothiocyanate (FITC)-labeled GPVI monomer or dimer in buffer containing 20 mmol/L HEPES pH 7.5, 50 mmol/L NaCl with 10 μL of the respective fibrin(ogen) or their proteolytic fragments serially diluted using the same buffer. The reactions were incubated at 4 °C for 5 minutes, followed by centrifugation at 17 000*g* to remove protein aggregates that may interfere with the measurement. The supernatants were loaded into standard treated capillaries before data collection using a Monolith NT.115 Series microscale thermophoresis instrument. The excitation/emission wavelength was set at 493/521 nm (blue channel). The LED (light-emitting diode) power was set at 75%, whereas 10% MST power was used for each binding experiment. Data were collected at 23 °C in 2 to 3 replicates. K_D_ was obtained by fitting the binding curve with the quadratic solution for the fraction of fluorescent molecules that formed the complex, calculated from the law of mass action (1:1 binding model).^27^ K_D_ was presented as mean±SD. When the binding curve was not saturated, the K_D_ was estimated as greater than the GPVI concentration that generates half of the maximum binding signal.

### GPVI-Fibrinogen/αC Fragments Binding and GPVI Intermolecular Interaction by Surface Plasmon Resonance

All SPR binding experiments were conducted using a Biacore 3000 instrument (GE Healthcare, Piscataway, NJ) using a binding buffer containing 10 mmol/L HEPES, pH 7.4, and 140 mmol/L NaCl, 0.05% tween 20. Biotinylated fibrinogen, monomeric, and dimeric GPVI were prepared by adding EZ-link NHS-LC-biotin dissolved in anhydrous dimethyl sulfoxide (DMSO) to the proteins dialyzed in PBS at a molar ratio of 2:1. The reactions were left on ice for 1 hour, followed by purification using NAP^TM^-5 desalting column, equilibrated with PBS, to remove excess unreacted biotin. Before ligand immobilization, streptavidin chips were conditioned with 3 consecutive 1-minute injections of 1 mol/L NaCl in 50 mmol/L NaOH at 30 μL/min. Biotinylated proteins were then diluted to 10 nmol/L and immobilized on streptavidin sensor chips at 5 μL/min in PBS buffer. Biotinylated fibrinogen, monomeric, and dimeric GPVI were immobilized on flow cell 2 (1500 response units [RU]), 3 (140 RU), and 4 (600 RU) respectively, whereas flow cell 1 was left without immobilized protein. The density of immobilized molecules on flow cell 3 and 4 are equivalent, given their different molecular weights. Monomeric and dimeric GPVI were flowed over at concentrations up to 20 μmol/L at a flow rate of 40 μL/min at 25 °C, by injecting 8 to 10 samples of the 2 proteins made from 2-fold serial dilutions plus 2 buffer injections. For GPVI-αC fragments interaction, the experiment was performed as detailed above, except that biotinylated αC fragments Aα 221 to 391, 368 to 610, and 221 to 610 were immobilized on flow cell 2 (80 RU), 3 (120 RU), and 4 (180 RU), respectively, and that only dimeric GPVI was used as analyte. The density of immobilized molecules on flow cell 2, 3, and 4 are equivalent, given their different molecular weights. Response signals obtained from flow cell 1 and buffer injections were subtracted in the data processing to obtain the final response curves. The data obtained from 5 lowest analyte concentrations where binding was observed were selected and fitted with 1:1 Langmuir model where association rate constant (*k*_*a*_), dissociation rate constant (*k*_*d*_), and dissociation constant (K_D_) were obtained.^28^ *k*_*a*_, *k*_*d*_, and K_D_ values were presented as mean±SD; N=3 for GPVI-fibrinogen interaction and N=2 for GPVI-αC fragments interaction. For GPVI-fibrinogen interaction, the highest analyte concentration curves were omitted in the fitting because they are less likely to follow the 1:1 interaction model than the lowest ones.

### AFM Imaging of GPVI/Fibrinogen Interactions

AFM images were taken using a surface of freshly cleaved mica that was treated with 50 μL of 2 mmol/L NiCl_2_ for 5 minutes. Following treatment, the surface was rinsed with deionized water and dried with nitrogen gas. For imaging of GPVI/fibrinogen interactions, GPVI monomer or dimer was incubated with fibrinogen at a 1:1 molar ratio for at least 2 hours in HBS. The solution was diluted to a final concentration of 2.5 μg/mL (8 nmol/L, fibrinogen) in HBS, added to the pretreated mica surface for 10 s, diluted with 50× deionized water for 10 s, and dried under nitrogen gas. High-resolution imaging was done using a Nanoscope IIIa Multimode AFM (Bruker, Santa Barbara, CA) in tapping mode with a scan rate of 0.8 Hz. All measurements were done in air using silicon cantilevers (TESPA-V2, Bruker Santa Barbara, CA) with a typical radius of 7 nm. Three to 5 images were taken from each sample and samples were repeated at least 3×. Standard flattening of images was performed.

For expression and purification of recombinant GPVI and fibrinogen/αC fragments, purification of plasma fibrinogen/αC fragments, preparation and purification of X-, D-, E-fragment and D-dimer, FITC labeling of GPVI, SD (sodium dodecyl sulfate-dithiothreitol) test, and additional SPR experiments, see supplemental methods.

### Statistical Analysis

For ELISA assays, all data were checked for normality by a Shapiro-Wilk test (alpha=0.05). Comparisons between related groups were performed by paired *t* test. Differences between samples and control (BSA or 0 nmol/L of GPVI-Fc/GPVI monomer) were compared using one-way ANOVA or Kruskal-Wallis test for multiple group comparisons, followed by Tukey-Kramer post hoc test of Dunn-Bonferroni to determine significance. Data analysis was performed using OriginPro 2017/2018 and Prism 8 GraphPad 2020. *P*<0.05 were considered to indicate statistical significance.

## Results

### Surface-Based Fibrinogen and GPVI Interaction

**Figure 1.**
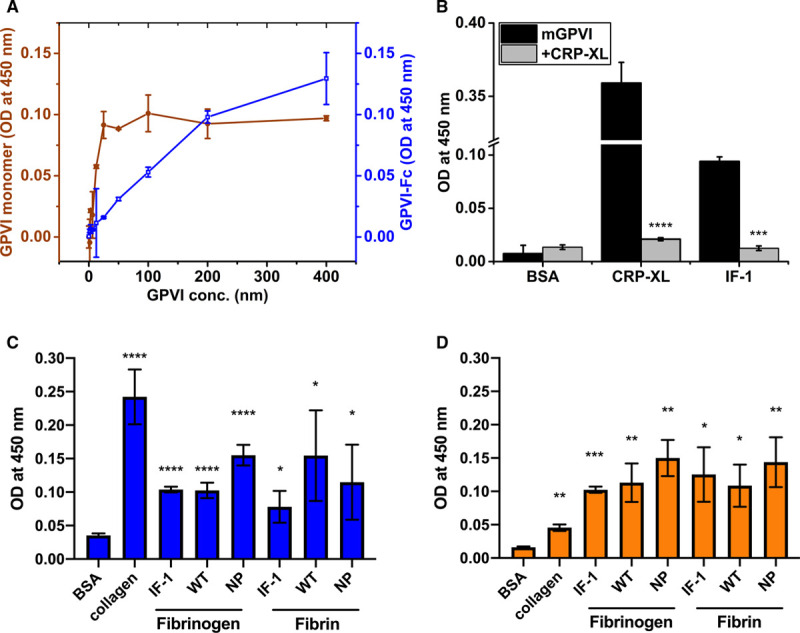
**Interaction of GPVI (glycoprotein VI)-Fc and GPVI monomer with immobilized fibrinogen measured by ELISA.** **A**, Concentration-dependent (0-400 nmol/L) binding of immobilized plasma-purified (IF-1) fibrinogen to GPVI-Fc (blue) and GPVI monomer (orange); (**B**) Immobilized CRP-XL (cross-linked collagen-related peptide) and IF-1 fibrinogen binding to 100 nmol/L GPVI monomer (black) and displacement of binding by 100 nmol/L CRP (gray); Binding of (**C**) GPVI-Fc dimer and (**D**) GPVI monomer to collagen, immobilized IF-1, recombinant wild-type (WT) and recombinant nonpolymerizing (NP) fibrinogen and fibrin (immobilized fibrinogen followed by cleavage by thrombin). Differences in optical density (OD) were compared between groups (**B**) or between immobilized ligand and BSA control (**C** and **D**); *P≤0.05, **P≤0.01, ***P≤0.001, ****P≤0.0001.

The binding of monomeric and dimeric GPVI to immobilized fibrinogen was analyzed by ELISA. Both forms showed concentration-dependent binding to fibrinogen (Figure 1A), although the GPVI monomer appeared to bind at lower concentrations than the dimer in this assay. It was further observed that binding between monomeric GPVI and fibrinogen could be displaced by preincubation with CRP-XL (Figure 1B), indicating that CRP-XL and fibrinogen likely share an overlapping binding site on GPVI. We next compared the ability of fibrinogen variants to bind GPVI, including plasma-purified, recombinant wild-type, nonpolymerizing-fibrinogen, and fibrin.^26^ Similar binding profiles to GPVI monomer and dimer were observed for all fibrinogen variants, suggesting that fibrin polymerization is not required for GPVI binding (Figure 1C dimer and 1D monomer). Indeed, when these fibrinogen variants were converted to fibrin through thrombin treatment, their binding profiles to GPVI remained similar (Figure 1C dimer and 1D monomer). We also observed that the dimeric form of GPVI appeared to partially displace the binding of GPVI monomer to immobilized fibrinogen, whereas addition of GPVI monomer had no impact on dimeric GPVI binding (Figure III in the Data Supplement). Binding of different types of fibrinogen to immobilized GPVI dimer and monomer was also observed (Figure III in the Data Supplement), confirming interaction of GPVI with fibrinogen in both orientations.

### GPVI Binds to Fibrinogen and CRP-XL in Solution

**Figure 2.**
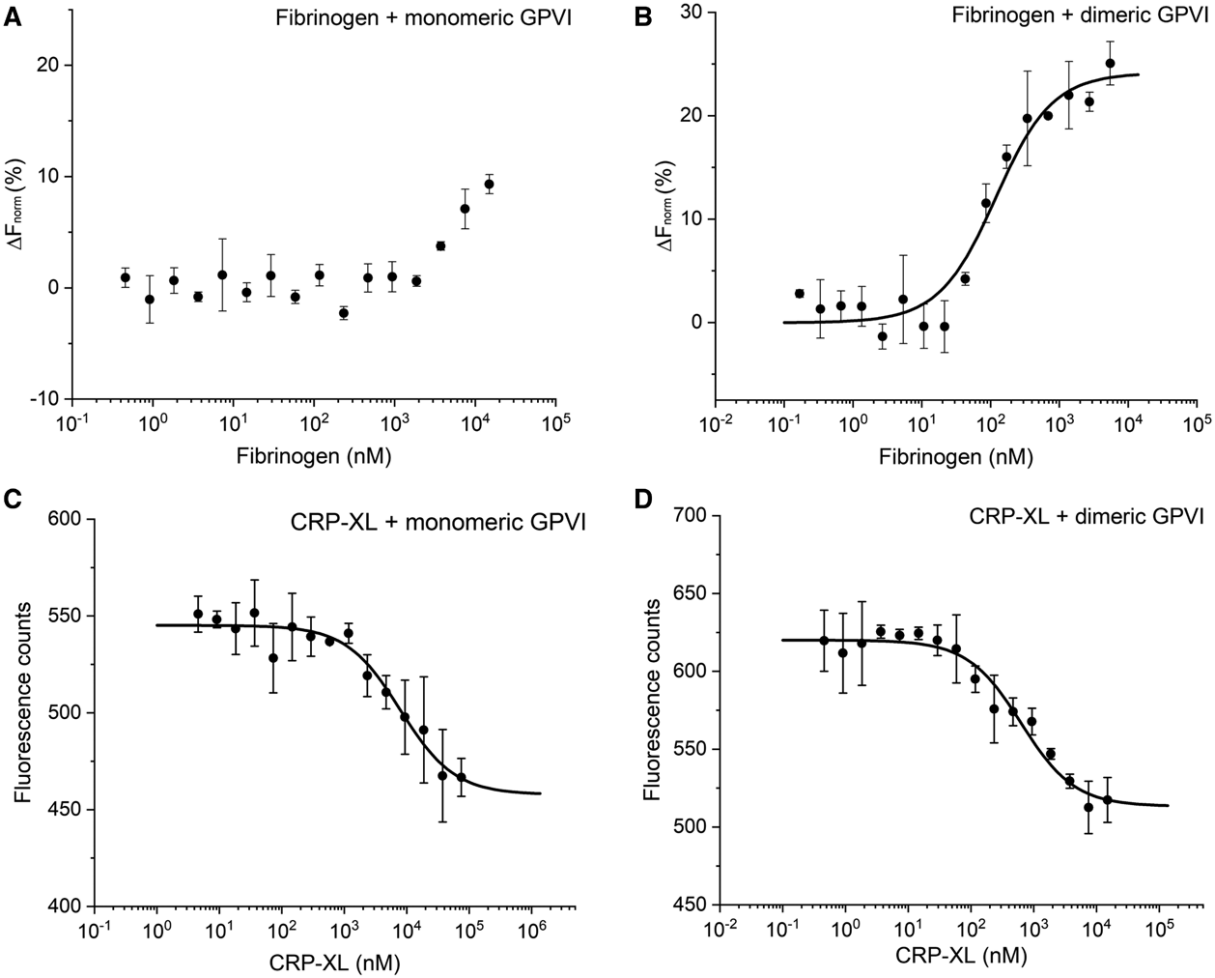
**GPVI (glycoprotein VI)-CRP-XL (cross-linked collagen-related peptide)/fibrinogen interaction analysis using microscale thermophoresis.** **A**, Fibrinogen binds to fluorescein isothiocyanate (FITC)-monomeric GPVI at K_D_ of >4 μmol/L, estimated as greater than the GPVI concentration that generates half of the maximum binding signal; (**B**) fibrinogen binds to FITC-dimeric GPVI at K_D_ of 99±2 nmol/L; (**C**) CRP-XL binds to FITC-monomeric GPVI at K_D_ of 5±0.8 μmol/L; (**D**) CRP-XL binds to FITC-dimeric GPVI at K_D_ of 603±207 nmol/L. Data were plotted using 3 independent replicates from which K_D_ values were obtained through fitting all the data with 1:1 interaction model using NanoTemper Analysis Software. K_D_ values are presented as mean±SD; N=3.

We next performed solution-based MST experiments by titrating fibrinogen to FITC-labeled GPVI monomer and dimer, respectively (Figure 2A and 2B). A saturable profile was observed for fibrinogen binding to dimeric GPVI, with a K_D1:1_ of 99±2 nmol/L. Interestingly, no saturation of binding was observed when titrating up to 10 μmol/L fibrinogen into monomeric GPVI. The K_D1:1_ of the latter interaction was estimated at >4 μmol/L (Table I in the Data Supplement). These data indicate that fibrinogen is able to bind both dimeric and monomeric GPVI in solution but with at least an order of magnitude higher affinity for dimeric than monomeric GPVI.

To analyze if GPVI binds to CRP-XL in solution by MST, we titrated CRP-XL into FITC-labeled monomeric and dimeric GPVI, respectively. As the CRP-XL concentration increased, the initial fluorescence intensity was reduced (Figure 2C and 2D), suggesting that CRP-XL interacts with both monomeric and dimeric GPVI, inducing quenching of the fluorescence. The fluorescence signal changes are specific to the binding of CRP-XL and were not caused by experimental artifacts, such as loss of fluorescent molecules due to surface absorption in which the fluorescence intensities of all samples were measured again after protein denaturation (see SD test and Figure IV in the Data Supplement). The binding data were fitted to a 1:1 stoichiometry model, resulting in a K_D1:1_=5±0.8 μmol/L and 603±207 nmol/L (hypothetical K_D_ calculated using the molar concentration of CRP-XL, monomeric equivalent), for the interaction of CRP-XL with monomeric and dimeric GPVI, respectively (Table I in the Data Supplement). Our observations that CRP-XL binds to dimeric GPVI more tightly than monomeric GPVI is in good agreement with the affinity data reported in the literature.^29–31^

### Surface Plasmon Resonance Analysis of Fibrinogen-GPVI Interaction

**Figure 3.**
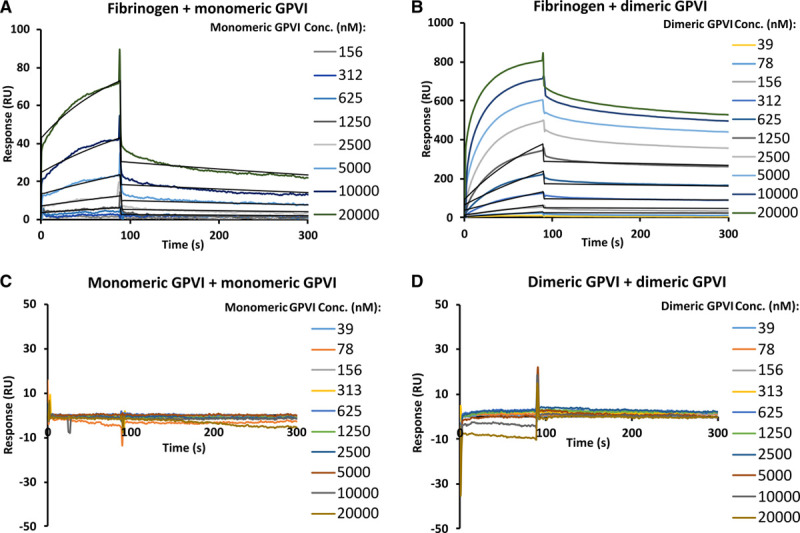
**GPVI (glycoprotein VI)-fibrinogen and GPVI intermolecular interaction analysis using surface plasmon resonance.** **A**, Monomeric GPVI binding to immobilized fibrinogen at K_D_ of 2.4±0.3 μmol/L; (**B**) dimeric GPVI binding to immobilized fibrinogen at K_D_ of 46±5 nmol/L. No apparent binding was observed for (**C**) monomeric GPVI binding to immobilized monomeric GPVI and (**D**) dimeric GPVI binding to immobilized dimeric GPVI. Data shown is one of 3 independent replicates from which K_D_ values were obtained through fitting individual data with Langmuir 1:1 interaction model. K_D_ values are presented as mean±SD; N=3. Conc. indicates concentration; and RU, response units.

To gain further insight into the kinetic profiles of GPVI-fibrinogen interactions, we performed SPR by immobilizing biotin-labeled fibrinogen as ligand on a streptavidin sensor chip with preimmobilized streptavidin. Monomeric and dimeric GPVI were flowed at various concentrations as analytes over the low-density fibrinogen surface at up to 20 μmol/L. Consistent with the MST data, distinct K_D1:1_ values of 2.4±0.3 μmol/L for monomeric and 46±5 nmol/L for dimeric GPVI (Figure 3A and 3B, Table II in the Data Supplement) were observed. Compared with monomeric GPVI, dimeric GPVI showed faster association rate constant than the monomer (8.5±0.2×10^3^ versus 5.7±0.2×10^2^ M^-1^S^-1^) and slower dissociation rate (6.1±0.1×10^-4^ versus 2.2±0.05×10^-3^ S^-1^; Table II in the Data Supplement). When GPVI dimer was flowed as analyte at 20 μmol/L, the response units were higher than that expected for 1:1 interaction (observed at 803 RU versus theoretical 1:1 interaction at 507 RU, Figure V in the Data Supplement). This observation suggests that dimeric GPVI interacts with >1 site on fibrinogen. To confirm the findings in ELISA that GPVI-fibrin(ogen) binding is not affected by fibrinogen polymerization, we immobilized nonpolymerizing-fibrinogen and converted it to fibrin using thrombin and determined their binding affinities with GPVI dimer using SPR. As observed for plasma-purified fibrinogen, nonpolymerizing-fibrinogen, and fibrin bound to GPVI dimer at a similar scale of K_D1:1_ (127±57 nmol/L and 86±21 nmol/L, respectively, Figure VI in the Data Supplement), which supports the above findings using ELISA. The observed slight decrease of binding affinity for nonpolymerizing compared with plasma-purified fibrinogen is possibly due to an observed slight α-chain degradation for nonpolymerizing-fibrinogen during expression in mammalian cells (Figure VII in the Data Supplement). Note that our data below have shown that this region of fibrinogen is crucial for GPVI binding (See Mapping Fibrinogen Regions for GPVI Binding Using Recombinant αC Fragments).

### GPVI Intermolecular Interactions

It has been reported that the 2 GPVI extracellular domains in GPVI-Fc (dimeric GPVI) may have a specific dimeric conformation that accounts for its stronger binding profile to collagen compared with monomeric GPVI.^29^ To gain further understanding on whether the 2 GPVI extracellular domains could dimerize to support this conformation, we designed SPR experiments where the intermolecular interactions of GPVI were measured by flowing monomeric and dimeric GPVI over immobilized monomeric and dimeric GPVI, respectively (Figure 3C and 3D). Our data show that no apparent binding was observed for either GPVI monomer to monomer or dimer to dimer. Similarly, the 2 proteins showed a single-peak gel filtration profile, with estimated molecular weights corresponding to single copies of their theoretical monomeric molecular weight (Figure VIII in the Data Supplement), indicating that they cannot dimerize or form stable higher oligomers in solution under these experimental conditions, consistent with the weak intermolecular contact observed in the crystal structure^30^ (Figure IX in the Data Supplement). Although intermolecular interaction is not detected under our experimental conditions, it is possible that at physiological conditions GPVI receptors on the cell surface may still dimerize or cluster upon platelet activation.^31–33^

### Mapping Fibrinogen Regions for GPVI Binding Using X-, D-, E-Fragment or D-Dimer

**Figure 4.**
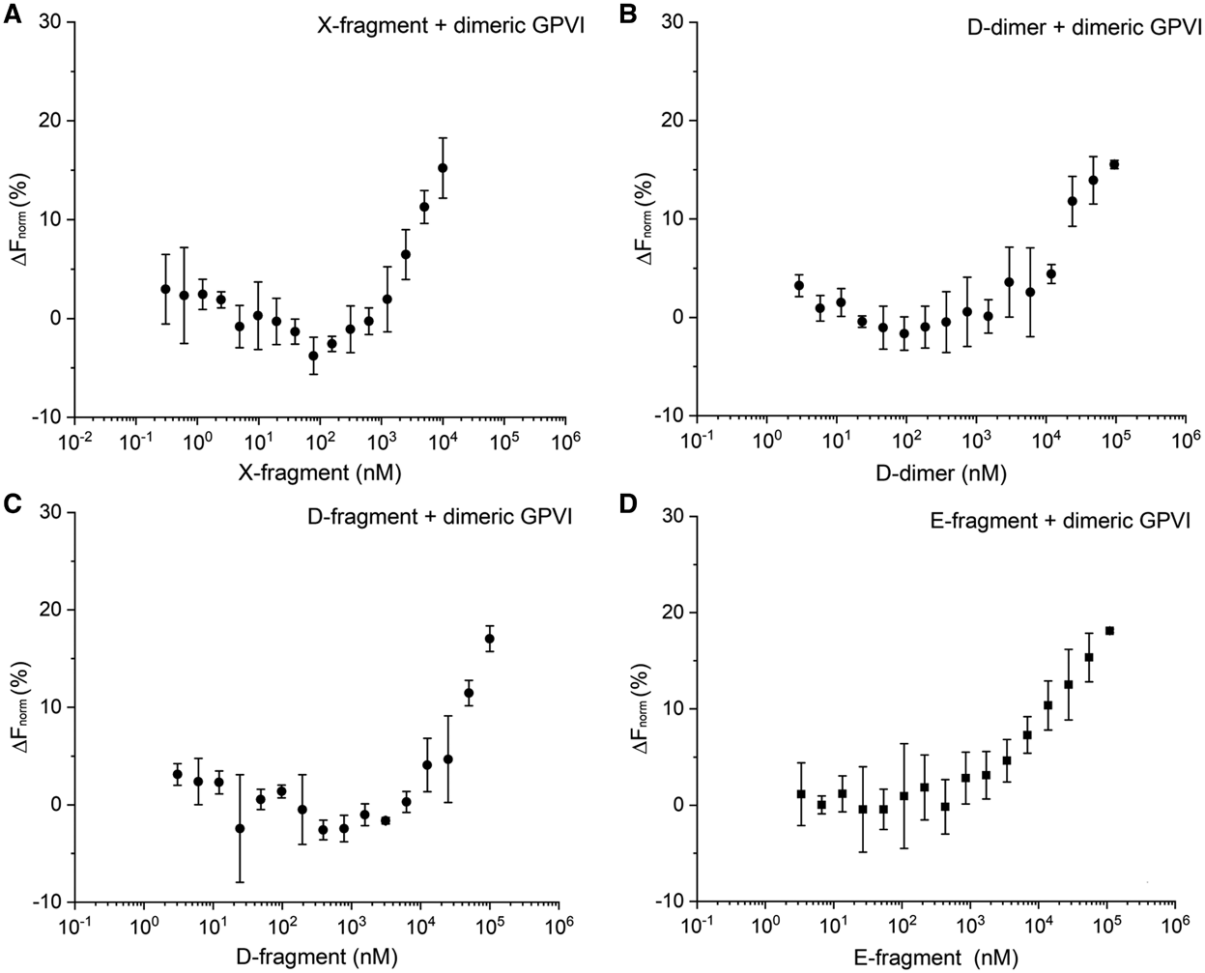
**Mapping GPVI (glycoprotein VI)-fibrinogen binding sites using microscale thermophoresis.** Binding profiles of (**A**) X-fragment, (**B**) D-dimer, (**C**) D-fragment, and (**D**) E-fragment to fluorescein isothiocyanate (FITC)-dimeric GPVI at K_D_ of >2 μmol/L for X-fragment and >20 μmol/L for D-, E-fragment and D-dimer. K_D_ values were estimated as greater than the GPVI concentration that generates half of the maximum binding signal. All results are presented as mean±SD; N=3 for **A–C** and N=2 for **D**.

**Figure 5.**
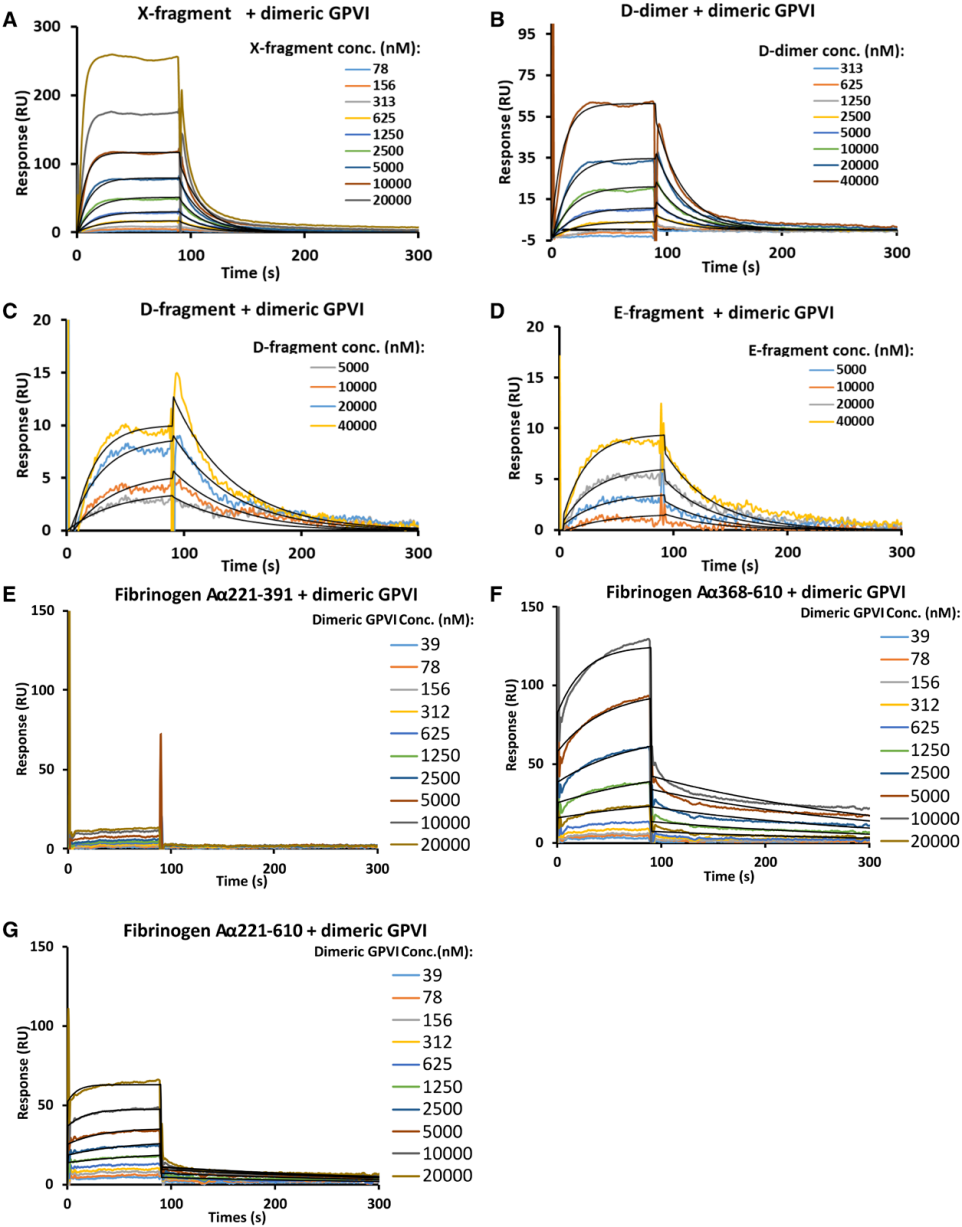
**Mapping GPVI (glycoprotein VI)-fibrinogen binding sites using surface plasmon resonance.** Binding profiles of (**A**) X-fragment, (**B**) D-dimer, (**C**) D-fragment, (**D**) E-fragment, (**E**) Aα 221-391, (**F**) Aα 368-610 and (**G**) Aα 221-610 to dimeric GPVI. No apparent binding was observed for Aα 221-391. Binding was observed for X-fragment, D-dimer, D-fragment, E-fragment, Aα 368-610 and Aα 221-610 at K_D_ of 9±1 μmol/L, 28±3 μmol/L, 26±5 μmol/L, 51±15 μmol/L, 2.3±0.2 μmol/L and 604±89 nmol/L, respectively. K_D_ values were determined through fitting individual data with Langmuir 1:1 interaction model (black lines). Each K_D_ value is the mean and SD of 3 replicates for **A–D** and 2 replicates for **F–G**. Conc. indicates concentration; and RU, response units.

To pinpoint regions of fibrin(ogen) that are responsible for GPVI binding, we produced proteolytic fragments of fibrinogen and fibrin for binding analysis with monomeric and dimeric GPVI (Figures IB and X in the Data Supplement). Binding affinities were first analyzed using MST. No binding was observed when 30 to 100 μmol/L X-, D-, E-fragment or D-dimer were titrated into monomeric GPVI (Figure XI in the Data Supplement). Although all the fragments did bind to dimeric GPVI, the binding profiles were not saturable at the fragment concentrations used (Figure 4A through 4D). The binding affinities were estimated at micromolar level (>2 μmol/L for X- fragment and >20 μmol/L for D-, E-fragment and D-dimer). We next performed SPR experiments to verify the findings from MST and obtain kinetic information of the interaction. When X-fragment and D-dimer were immobilized as ligands, no clear bindings were observed for GPVI monomer and dimer at 1 and 10 μmol/L, respectively (Figure XII in the Data Supplement). Binding was detected at a reversed orientation (immobilizing monomeric and dimeric GPVI as ligands and flow over fibrin(ogen), X-, D-, E-fragment or D-dimer as analyte). Although both monomeric and dimeric GPVI bound to fibrin(ogen) fragments, binding was not saturable at 40 μmol/L analytes concentration (Figure XIII in the Data Supplement, Figure 5A through 5D). The kinetics of binding between monomeric and dimeric GPVI to the fibrin(ogen) fragments differed from each other, with monomeric GPVI having more rapid association and dissociation rate constants than the dimer. This could explain why binding was not observed in MST for those proteins, as their rapid off rates suggest that a stable complex is not likely formed and, therefore, not detected in MST. The K_D_ was obtained for dimeric GPVI binding to X-fragment, D-dimer, D-fragment, and E-fragment at 9±1, 28±3, 26±5, and 51±15 μmol/L, respectively (Table II in the Data Supplement). Due to the very rapid association and dissociation rates, the K_D_ for monomeric GPVI binding to all the fragments could not be determined through fitting the kinetic data. The affinities were estimated from a simulated SPR sensorgram similar to that observed by modifying the on and off rates for dimeric GPVI-X-fragment interaction. The simulation revealed that the K_D_ for monomeric GPVI-X-fragment interaction is at least >128-fold higher, at >1 mmol/L (Figure XIV in the Data Supplement), than that observed for dimeric GPVI-X-fragment interaction (9±1 μmol/L). These data indicate that fibrinogen fragments interact with monomeric and dimeric GPVI with distinct affinities and kinetic profiles. Compared with the binding data obtained using full-length fibrinogen, which showed nano-molar affinity for GPVI, the data indirectly show a major role of the fibrinogen αC-region in GPVI binding, which is not present in any of the proteolytic fragments, but normally present in full-length fibrinogen.

### Mapping Fibrinogen Regions for GPVI Binding Using Recombinant αC Fragments

To confirm the proposed major role of fibrinogen αC-region in GPVI binding and identify specific binding regions on αC for GPVI recognition, we expressed 3 recombinant fibrinogen αC fragments in *Escherichia* *coli*: full-length (Aα 221-610); N-terminal linker (Aα 221-391) and C-terminal globular domain (Aα 368-610)^34–37^ and purified them to homogeneity (Figure XV in the Data Supplement). These fragments were immobilized, and binding affinities with GPVI dimer were quantified using SPR. No apparent binding was found when up to 20 μmol/L of GPVI dimer was flowed over the N-terminal linker (Figure 5E). Binding was observed for the full-length and C-terminal globular domain to GPVI dimer, at 604±89 nmol/L and 2.3±0.2 μmol/L, respectively (Figure 5F through 5G, Table II in the Data Supplement). These data directly show that αC-region is playing a more important role than other fibrinogen regions in GPVI-fibrinogen interaction.

### Molecular Imaging of GPVI-Fibrinogen Interactions

**Figure 6.**
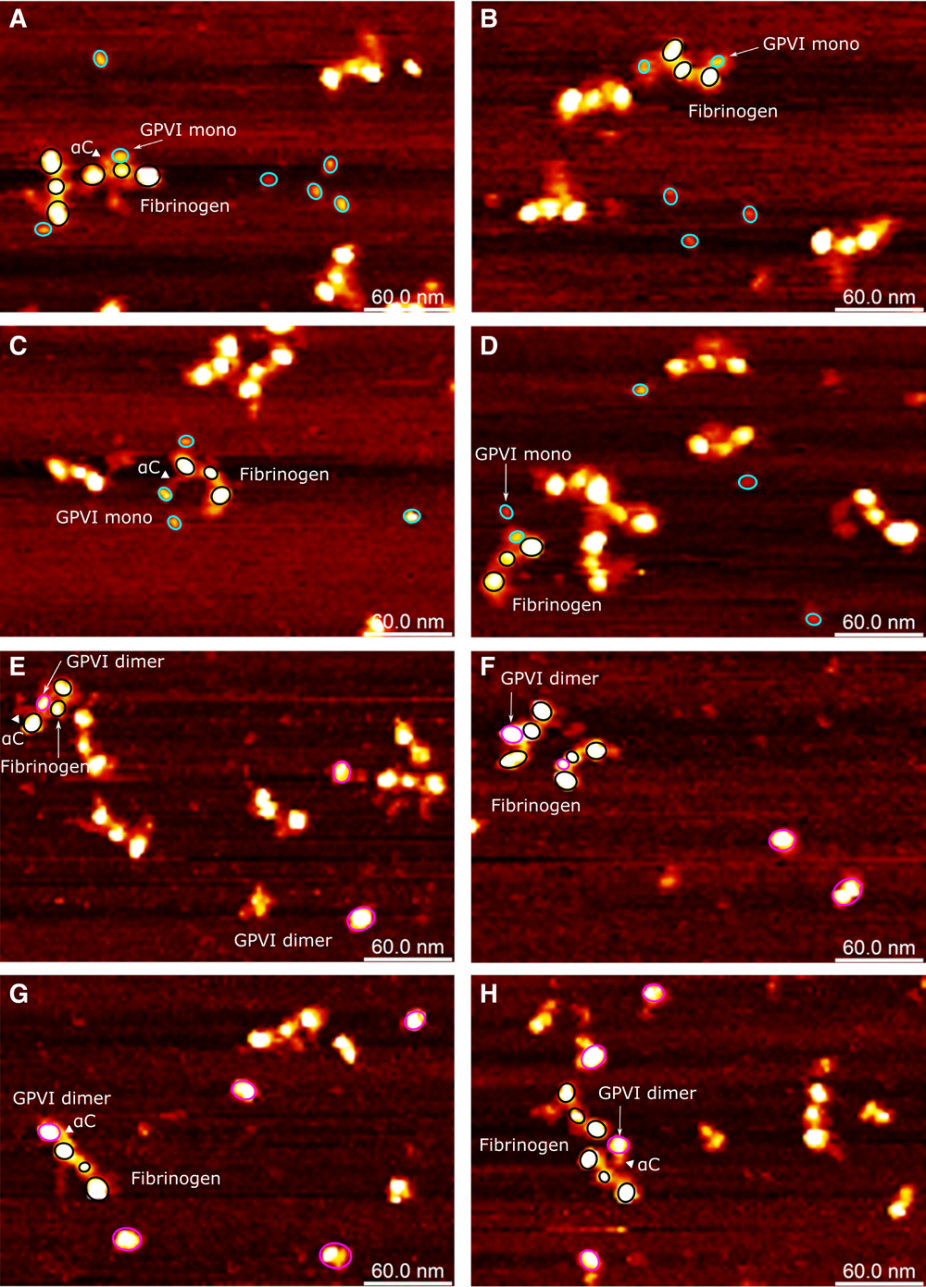
**Atomic force microscopy topography images of interactions between GPVI (glycoprotein VI) and fibrinogen.** The fibrinogen molecules are highlighted using 3 black circles for its D-E-D structure. The disordered fibrinogen αC-regions are visible as flexible appendices with reduced height (and therefore reduced brightness) from the D-region or close to the E-region. **A–D**, GPVI monomer are indicated with cyan circles; (**E–H**) GPVI dimer are indicated with magenta circles. The αC-region of fibrinogen involved in GPVI binding is highlighted with triangles. Two structural isoforms of GPVI dimer were observed: one as a large single-domain globular protein and the other as an elongated shape or as 2 domains with similar sizes closely associated with each other.

To verify our observations from MST and SPR, and to gain further understanding of the structural regions of fibrinogen that GPVI may interact with, we performed AFM by mixing the 2 proteins and directly visualizing the interactions occurring and the resulting protein complexes (Figure 6). Monomeric GPVI appeared as an elongated globular structure (Figure 6A through 6D). The dimeric GPVI particles were observed adopting 2 different forms, single nodular or binodular (Figure 6E through 6H). Given that the intermolecular interaction is very weak as observed in SPR (Figure 3C through 3D), it is likely that the single and binodular forms represent top and side views of the GPVI dimer molecule. In the side view, the GPVI part and Fc domain should be visualized as 2 nodules closely attached. In the top view, the GPVI part and Fc domain should overlap, visualized as a single nodule. Fibrinogen on the AFM images is readily recognizable as a typical trinodular protein (D-E-D) with the highly flexible and disordered αC-regions visibly extending from the D-region. The αC-region can extend as far as the E-region and interact with this part of the molecule.^38,39^ Both GPVI proteins generated complexes with fibrinogen, by apparently interacting with the αC-region through different topologies based on the disordered nature of this region of the fibrinogen molecule (Figure 6A, 6C, 6E, 6G, and 6H). These data are consistent with the SPR and MST data showing an order of magnitude drop in the affinity of GPVI when comparing binding to full-length fibrinogen with the X-fragment lacking the αC-region, and the direct binding of recombinant full-length and C terminus of the fibrinogen αC-region to GPVI dimer shown by SPR, indicating that the αC-region is critical for the interaction of GPVI with fibrinogen.

## Discussion

Our study provides key molecular insights into the interaction of GPVI with fibrinogen, with 2 main central findings. First, we found that fibrinogen interacts with monomeric and dimeric GPVI via 2 distinct affinities and kinetics profiles. The higher affinity of dimeric GPVI for fibrinogen is likely due to increased avidity, based on the observations that binding to dimeric GPVI has slower dissociation kinetics than the monomer, and that no intermolecular interactions were found for GPVI. Second, our data show the αC-region of fibrinogen as the key binding region for GPVI interaction, indicating that the αC-region is playing a more important role than any other fibrinogen regions for GPVI binding.

Previous studies reported similar affinities for the binding of GPVI to fibrinogen and fibrin, indicating that fibrin polymerization is not required for GPVI binding.^20–22,25^ In agreement with this, we observed no differences in GPVI-fibrinogen and GPVI-fibrin binding profiles by ELISA. Furthermore, we obtained similar binding profiles with our new fibrin mutant that is unable to polymerize^26^ when compared with polymerized fibrin preparations, confirming that polymerization of fibrinogen into fibrin is not required to elicit GPVI binding. Although GPVI is able to bind both fibrinogen and fibrin, we hypothesize that platelet activation only occurs when GPVI is clustered, which should happen if fibrin is polymerized into fibers or film,^40^ but not with fibrinogen in solution. Platelet activation via GPVI-fibrinogen interaction should also occur when platelets are interacting with fibrinogen coated to a plate because the close proximity of fibrinogen coated to the surface will support clustering of GPVI on the platelet. This is supported by differences observed in GPVI-deficient platelet spreading on fibrinogen-coated surfaces compared with normal platelets.^19^

Our MST and SPR data suggest that although both monomeric and dimeric GPVI can interact with fibrinogen, dimeric GPVI showed higher affinity for fibrinogen than the monomer with a slower dissociation rate. Similar observations have also been made for other receptor-ligand interactions, such as Eph-ephrins (Fc-fused) and KIR2D-HLA-G (killer immunoglobulin-like receptor 2D-human leukocyte antigen G; disulfide-linked), as well as for engineered binding protein-ligand interactions, such as affibodies and bispecific antibodies.^41–47^ The increased affinity for the fused dimer to their binding partner accompanied by a slower dissociation rate over the monomer could be explained by the avidity effect. These findings are reminiscent of our observations for GPVI-fibrinogen interaction, as well as those reported for GPVI-collagen interaction.^29^ It is likely that the affinity increase with a slower dissociation rate for the GPVI dimer compared with the monomer when binding to fibrinogen can also be attributed to the similar avidity effect mediated by the increased valency in the interaction.^48^

Our SPR analysis of GPVI intermolecular interaction where no self-assembly was observed implies that the increased affinity for dimeric GPVI binding to fibrinogen is more likely dominated by avidity effects than a dimeric GPVI conformation. Similar avidity model may also be applicable to other multivalent GPVI ligands, such as collagen, where distinct apparent affinities were also observed when interacting with GPVI monomer and dimer.^29^ Nevertheless, the conditions in which the intramolecular interaction was analyzed in our study are different than those occurring physiologically, where the GPVI is presented with all parts of the receptor including the stalk and cytoplasmic tail. Furthermore, the localization of GPVI on the platelet surface may facilitate dimerization, which cannot be achieved under solution conditions. Therefore, it cannot be ruled out that GPVI receptors on the platelet surface could form a specific and functional dimer conformation when interacting with these ligands. The GPVI dimer conformation is supported by several reports using GPVI dimeric specific antibodies and Fabs showing the presence of GPVI dimers on the platelet surface upon platelet activation.^33,49–51^ More in-depth studies utilizing structural approaches, such as Cryo-EM (cryogenic electron microscopy), crystallography, and NMR (nuclear magnetic resonance), are needed to elucidate the molecular and conformational detail of the complete GPVI receptor on platelet surface and how this underpins interactions with its various biological ligands.

Whether the monomeric or dimeric GPVI binds to fibrin(ogen) at high affinity has been a conflicting topic debated in the literature.^19,22,23^ Several possible reasons to account for the apparently conflicting results have been proposed, such as variations in GPVI constructs and binding assay conditions among different labs.^25^ Another important factor that could contribute to the confliction is the limitations of ELISAs.^19,21,22^ For example, nonspecific signals could be induced through cross-reactivity of the multiple protein components used in the assays.^52^ Differences in affinity of antibodies to either monomeric or dimeric GPVI could also play a role in the discrepancies by producing different levels of signal, which is indirectly related to both the affinities of the antibodies as well as the affinities of GPVI for the ligands. More direct methods, such as SPR and MST, are needed to support these results. Another reason for the discrepancies in previous studies is the source and quality of the fibrinogen used. The αC-region that appears to contribute most of the binding affinity is susceptible to protein degradation and highly variable in different fibrinogen preparations. All the above are likely responsible for some of the discrepancies reported in the literature.

Our data show that the fibrinogen αC-region, which has not yet previously been implicated in GPVI binding, plays an important role in interactions with GPVI, as shown by the observed direct interaction of recombinant αC fragments with GPVI and decreased binding affinity after removing the αC-region from fibrinogen in the X-fragment. Our data further suggest that the GPVI binding region lies in the C-terminal globular domain of the αC-region, by comparing the binding profiles of the full-length, N and C terminus of the αC. Interestingly, the C terminus of the αC-region binds to GPVI dimer at a lower affinity than the full-length αC fragment and fibrinogen, indicating that binding is likely stabilized by other parts of the fibrinogen molecule, consistent with the disordered nature of the αC-region. The fibrinogen αC-region contains several important binding sites for integrin receptors such as αIIbβ3,^53^ αvβ3,^54^ and αvβ1.^55^ Moreover, it also interacts with several plasma proteins including FXIIIa,^34^ fibronectin,^56^ apolipoprotein,^57^ plasminogen,^35^ tPA (tissue-type plasminogen activator),^35^ and α2-antiplasmin,^58^ each of which plays important roles in hemostasis, thrombolysis and other vascular processes. The αC-region proves to play a key role in the interaction of fibrinogen with other proteins, leaving the D- and the E-regions available and solely accessible to drive fibrin polymerization through knob-hole binding. The αC-region extensions from the D-region thus act as long flexible tethers that serve the purpose of bringing other proteins with important vascular functions into close contact with the generating fibrin network during blood clotting.

In conclusion, our data support that avidity is responsible for the higher affinity of dimeric GPVI binding to fibrinogen over the monomer, mediated through increased valency of interaction. Avidity-based mechanisms have been suggested for similar protein interaction systems and, therefore, could be utilized by other multivalent GPVI ligands, such as collagen and anti-GPVI antibodies when interacting with GPVI. These observations provide new insight into our understanding of the mechanism of GPVI interactions with its multivalent ligands and clarify some of the discrepancies reported in the literature, explained by the limitations of ELISAs. Our analysis also suggests that the αC-region of fibrinogen is an essential and important region for fibrinogen-GPVI interaction, which shed light on how the interaction of fibrinogen and fibrin with GPVI receptor on the surface of platelet impact thrombosis. When coagulation leads to the generation of fibrin and fibrin fibers, interaction with the fibers that are being generated clusters GPVI on the platelet surface (Graphical Abstract), leading to rapid signaling, platelet activation, and sustained blood clot growth away from the collagen surface that initially triggered clot formation at the site of injury. Future work should focus on pinpointing the exact binding motif for GPVI on the fibrin(ogen) αC-region. The outcome from such studies could contribute to the development of novel and safer antithrombotic inhibitor drugs targeting GPVI-fibrin(ogen) interaction with the fibrin(ogen) αC-region.

## Acknowledgments

We gratefully acknowledge the provision of GPVI (glycoprotein VI) proteins and expression constructs by Dr Andrew Herr, Cincinnati Children’s Hospital.

## Sources of Funding

This work was supported by a joint Welcome Trust Investigator Award (204951/B/16/Z) to S.P. Watson and R.A.S. Ariëns. Surface plasmon resonance (SPR) and microscale thermophoresis (MST) binding assays were performed in the Biomolecular Interactions facility, Astbury Centre for Structural Molecular Biology, Faculty of Biological Sciences, University of Leeds (part-funded by the Wellcome Trust 062164/Z00/Z). S.P. Watson holds a British Heart Foundation (BHF) Chair (03/003).

## Disclosures

None.

## Supplementary Material


