# Supplementary material for: GPVI (Glycoprotein VI) Interaction With Fibrinogen Is Mediated by Avidity and the Fibrinogen αC-Region
Source: Arterioscler Thromb Vasc Biol. 2021 Jan 21;41(3):1092–104. doi: 10.1161/ATVBAHA.120.315030 (PMC7901536; doi:10.1161/ATVBAHA.120.315030)
Supplement: Supplementary file 1 [file atv-41-1092-s001.pdf]

GPVI interaction with fibrinogen is mediated by avidity and the fibrinogen αC-region

Rui-Gang Xu[1], Julia S. Gauer[1], Stephen R. Baker[1,2], Alexandre Slater[3], Eleyna M. Martin[3], Helen R. McPherson[1], Cédric Duval[1], Iain W. Manfield[4], Arkadiusz M. Bonna[5], Steve Watson[3], Robert A. S. Ariëns[1*]

[1] Discovery and Translational Science Department, Institute of Cardiovascular and Metabolic Medicine, University of Leeds, Leeds, LS2 9LU, UK.

[2] Department of Physics, Wake Forest University, Winston Salem, NC 27103, USA

[3] Institute of Cardiovascular Sciences, College of Medical and Dental Sciences, University of Birmingham, Birmingham, B15 2TT, UK.

[4] School of Molecular and Cellular Biology, Faculty of Biological Sciences, University of Leeds, Leeds, LS2 9LU, UK.

[5] Department of Biochemistry, University of Cambridge, CB2 1GA, UK.

[*]Corresponding Author: Robert A. S. Ariëns, Discovery and Translational Science Department, Institute of Cardiovascular and Metabolic Medicine, University of Leeds, LS2 9LU, UK. Tel: +44 113 3437734. Email: R.A.S.Ariens@leeds.ac.uk

Supplemental Methods:

The data that support the findings of this study are available from the corresponding author upon reasonable request.

Expression and purification of recombinant GPVI

GPVI-Fc construct was expressed and purified, as previously described[1]. In short, GPVI residues S2-T183 were sub-cloned into SigpIg+ mammalian expression vector between a CD33 signal sequence (N-terminus) and a FXa cleavage site followed by a human IgG1-Fc sequence (C-terminus) (kindly provided by Dr. Andrew Herr, Cincinnati Children's Hospital, USA). GPVI-Fc was expressed by transient transfection of HEK293T cells with polyethylenimine hydrochloride. Cell culture medium containing secreted protein was purified by protein-A affinity chromatography using HiTrap MabSelect, with eluted proteins further purified by gel filtration in HBS (10 mM HEPES, pH 7.4, 140 mM NaCl) using Superdex S200 10/300 Increase. Purified GPVI-Fc was cleaved overnight with FXa to produce monomeric GPVI, which was further purified from the cleavage product by gel filtration. An alternative construct, comprised of residues S2-T182 with a hexahistidine tag at the C-terminus, was inserted into the eukaryotic expression vector pEE12.4 by HindIII/EcoRI restriction endonuclease digestion and subsequent T4 DNA ligase. This construct was expressed as described above and purified by Ni-NTA to produce monomeric GPVI-His tagged, which was used in all enzyme-linked immunosorbent assays.

Expression of recombinant WT/NP fibrinogen[2]

Recombinant human wild-type (WT) and non-polymerising AαR19S/BβR17S (NP) fibrinogen expression was performed as previously described[3, 4]. Briefly, expression vectors pSELECT-Zeo and pSELECT-Puro (Invivogen, Toulouse, France) containing the cDNA sequences of α- or β-chain were mutated at codons 19 and 17, respectively, using QuickChange II Site-Directed Mutagenesis Kit. Mutations were confirmed by sequencing (MRC Protein Phosphorylation and Ubiquitylation Unit DNA Sequencing Services, Dundee, UK). Chinese hamster ovary (CHO) cells containing the γ-chain were transfected with the mutated plasmids. WT and NP fibrinogens were produced in roller bottles coated with adherent microcarrier beads, containing DMEM/F12 medium supplemented with 2 mg/ml aprotinin, 5 µg/mL transferrin, 5 µg/mL insulin and 5 ng/mL sodium selenite. Medium was collected every 48 hours and stored at -40 °C with the addition of 150 µM PMSF for a period of 8 weeks. Fibrinogen was precipitated from harvested cell culture medium overnight with 40% saturated ammonium sulphate and a protein inhibitors mixture containing 20 mM MES hydrate pH 5.6, 5 mM 6-aminohexanoic acid, 5 mM benzamidine, 100 µM PMSF, 1 µM leupeptin and 1 µM pepstatin). Following precipitation, the medium was centrifuged at 14,500 *g* at 4 °C for 45 min without brakes. The pellet was re-suspended in 333 mM NaCl, 222 mM Tris, 111 µM PMSF, 5 µM pepstatin, 5 µM leupeptin, 1 mM EDTA, 11 U/mL trypsin inhibitor, 5 mM benzamidine and 5 mM 6-aminihexanoic acid, and incubated at 4 °C for 30 min prior to centrifugation at 43,000 *g* for 30 min. Supernatant was collected and stored at -80 °C before purification by IF-1 immunoaffinity chromatography, using buffers

described in the next section. $CaCl_2$, to a final concentration of 10 mM, was added to samples prior to chromatography. Fractions containing fibrinogen were collected and stored at -80 °C before concentration and dialysis in TBS (50 mM Tris-HCl and 100 mM NaCl pH 7.4). Protein concentration was determined at A_{280nm} (extinction coefficient: $E_{1\%}$ = 15.1), and protein integrity and purity were assessed by SDS-PAGE under reducing conditions.

Expression of recombinant fibrinogen αC fragments

Fibrinogen αC fragments Aα 221-391, 368-610 and 221-610 were amplified by PCR using the cDNA coding for full length fibrinogen Aα-chain as template[2] The amplified inserts were ligated into the pCR2.1 vector using TOPO-TA cloning kits to allow for better cleavage of restriction sites. This was followed by digestion with BamHI and SalI and ligation into the pGEX-6P-1 vector. The coding sequence of the Aα constructs was confirmed by DNA sequencing. The Aα fragments were expressed as previously described[5]. Briefly, the three constructs were transformed into BL21-Gold competent cells for protein expression. The cells were grown at 37 °C in TB broth until the OD600 reached 1.0. The cells were induced by 1 mM IPTG and the culture was incubated at 31 °C for 16h. The cultures were centrifuged at 5,000 g for 10 min and the pellets resuspended in ice-cold PBS buffer. The resuspended cultures were centrifuged at 4,500 g for 30 min at 4°C and the pelleted cells frozen at -20°C. Harvested cells were resuspended in PBS buffer with 1 mg/ml lysozyme, 1 mM DTT, 2 μg/ml Aprotinin, 1 μM Pepstatin A, 10 μM Leupeptin, 4 mM Benzamidine and 0.5 mM PMSF. The lysate was sonicated on ice for 10 cycles (sonicate for 2 min and rest for 1 min as per cycle). After sonication, 20% Triton X-100 was added to the lysate at 1% final concentration to aid solubilisation of fusion protein, followed by centrifugation at 22,000g for 20 min. The supernatant was pooled and filtered with 0.22 μm syringe filters. GST fusion proteins were purified by GST-affinity chromatography using an AKTA Pure system according to the recommended method by GE healthcare. Briefly, filtered lysate was loaded onto the column equilibrated with PBS buffer overnight. Column was washed with PBS buffer containing 0.2% Triton X-100, followed by manual injection of PreScission™ Protease (at 2 U per 100 μg of protein following the recommended protocol for on-column GST cleavage by GE healthcare) and incubation at 4 °C overnight. The GST-tag cleaved αC fragments were eluted with a buffer containing 50 mM Tris-HCl and 10 mM reduced glutathione, pH 8.0. The fractions containing cleavage proteins were confirmed by SDS-PAGE, concentrated, and further purified using a S200 10/300 Increase gel filtration column, equilibrated with 20 mM HEPES, 150 mM NaCl, pH 7.5 for Aα 221-391 and Aα 221-610. For Aα 368-610 a buffer containing 20 mM HEPES, 1 M NaCl, pH 7.5 was used instead to facilitate separation of non-specifically bound DNA from the protein. The fractions containing target proteins were confirmed by SDS-PAGE, concentrated, and stored at -80 °C.

Plasma purified fibrinogen

Plasminogen-depleted human fibrinogen was further purified by IF-1 immunoaffinity chromatography. Briefly, fibrinogen at 10 mg/ml in TBS and 1 mM $CaCl_2$ was loaded

onto to the column, equilibrated with 50 mM Tris pH 7.4, 300 mM NaCl and 1 mM $CaCl_2$. The column was washed with 50 mM Tris pH 7.4, 1 M NaCl and 1 mM $CaCl_2$, followed by a wash with 50 mM NaAc pH 6.0, 300 mM NaCl and 1mM $CaCl_2$. The protein was eluted with 50 mM Tris pH 7.4, 300 mM NaCl and 5 mM EDTA. Peak fractions containing fibrinogen were pooled and dialysed into TBS before being snap frozen in liquid nitrogen and stored at -80 °C.

Fibrinogen X-fragment

Human fibrinogen was dissolved in TBS to a final concentration of 2 mg/ml, followed by the addition of bovine trypsin (final concentration 12.5 µg/ml). The mixture was incubated at room temperature for 30 min, followed by the addition of soybean trypsin inhibitor to a final concentration of 25 µg/ml. The mixture was concentrated and kept at 4 °C before separation of the X-fragment using size-exclusion chromatography on a S200 16/60 column equilibrated with HBS. Peak fractions containing purified X-fragment were pooled, snap frozen in liquid nitrogen and stored at -80 °C.

Fibrinogen D-fragment

Human fibrinogen was dissolved in TBS to a final concentration of 2 mg/ml, followed by adding 5 mM EDTA and bovine trypsin to a final concentration of 25 µg/ml. The mixture was incubated at room temperature for 2 h, followed by the addition of soybean trypsin inhibitor to a final concentration of 50 µg/ml. The mixture was then concentrated and kept at 4 °C before separation following the same size-exclusion procedure mentioned for the X-fragment.

Fibrin D-dimer and E-fragment

Human fibrinogen dissolved in TBS at 2 mg/ml was clotted at room temperature for 30 min by adding bovine thrombin and recombinant FXIII-A to a final concentration of 0.4 U/ml and 2 µg/ml, respectively. The clot was fragmented into small pieces using a spatula, followed by the addition of bovine trypsin to a final concentration of 25 µg/ml. The reaction was incubated at room temperature for 2 h, before the addition of soybean trypsin inhibitor to a final concentration of 50 µg/ml. The mixture was then concentrated and kept at 4 °C before purification following the same size-exclusion protocol mentioned for the X-fragment.

FITC labelling of recombinant GPVI

For protein labelling, GPVI-Fc and monomer concentration were adjusted to 1 mg/ml using PBS (137 mM NaCl, 2.7 mM KCl, 8 mM Na_2HPO_4, and 2 mM KH_2PO_4, pH 7.4). The solid FITC dye was dissolved in 100% anhydrous DMSO at a concentration of 1.3 mM and mixed thoroughly. Before mixing, the concentration of the dye was adjusted to 2-3-fold concentration of the protein using the labelling buffer. Then, the protein and the fluorescent dye solutions were mixed in 1:1 ratio and incubated for 60 min on ice in the dark. Unreacted dye was eliminated by NAP-5 columns. The degree of labelling was determined by measuring fluorophore absorption at 495 nm and the protein absorbance at 280 nm, corrected for the FITC fluorophore.

SD test

In circumstances where the initial fluorescence in the capillary scan changes over the increasing protein concentrations and deviates from the base line by more than 20%, SD test were performed to verify if this fluorescence change is induced by protein-protein interaction or other effects such as protein absorption to the capillary. For the SD test, a 2x SD solution containing 40 mM DTT and 4% SDS was mixed with equal volume of the 16 reactions. Samples were denatured by heating at 95 °C for 5 min and centrifuged to ensure all the sample is at the bottom of the tube. The initial fluorescence of the denatured samples was measured following the same procedure as used for the un-denatured samples. If the fluorescence intensities for all samples are similar after the SD test, it can be concluded that the previously observed fluorescence changes were induced by a binding event. In this analysis, the fluorescence intensities for all samples were similar (Fig IV), indicating that the observed reduction in intensity was due to CRP-XL binding to FITC-labelled GPVI.

Immobilised GPVI interaction with fibrin(ogen) fragments binding by surface plasmon resonance

Monomeric and dimeric GPVI were immobilised on the CM-5 chip surface using amine-coupling, as described above. Briefly, immobilisation was carried out using the monomeric and dimeric GPVI proteins diluted at 5 µg/ml in 100 mM NaAc, pH 5.6 under a flow rate of 5 µl/min. Flow cell 1 was made as a mock derivatisation. Monomeric GPVI was immobilized on flow cell 2 to a final density of 140 RU and dimeric GPVI was immobilised in flow cell 3 to a final density of 600RU. Fibrinogen X-fragment, D-fragment, fibrin D-dimer and E-fragment were flowed over at concentrations up to 40 µM at a flow rate of 40 µl/min at 25°C, by injecting 8 samples of each fibrinogen fragment made from two-fold serial dilutions plus two buffer injections. Data analysis was performed as mentioned above.

GPVI interaction with immobilised fibrin(ogen) fragments binding by surface plasmon resonance

E-fragment, D-dimer and X-fragment were immobilised on the CM-5 chip surface using amine-coupling. Samples were diluted at 5 µg/ml in 100 mM NaAc, pH 5.6 under a flow rate of 5 µl/min and immobilised on flow cell 2 (180RU), 3 (700RU), and 4 (1080RU) respectively, whereas flow cell 1 was left without immobilised protein. The density of immobilised molecules on flow cell 2, 3 and 4 are equivalent. Monomeric and dimeric GPVI were flowed over at concentrations up to 1 and 10 µM respectively, at a flow rate of 40 µl/min at 25 °C, by injecting 2 samples from ten-fold dilutions plus a buffer injection. Response signals obtained from flow cell 1 and buffer injections were subtracted in the data processing to obtain the final response curves.

GPVI interaction with immobilised NP-fibrin(ogen) fragments binding by surface plasmon resonance

Biotinylated NP-fibrinogen were prepared by adding EZ-link^{TM} NHS-LC-biotin dissolved in anhydrous DMSO to the proteins dialysed in PBS at a molar ratio of 2:1. The reactions were left on ice for 2 h, followed by purification using NAP-5 desalting column, equilibrated with PBS, to remove excess amount of unreacted biotin. Biotinylated proteins were diluted to 10 nM and immobilised on a Streptavidin (SA) sensor chips at 5 µl/min in PBS buffer. Before ligand immobilisation, SA chip was conditioned with 3 consecutive 1-minute injections of 1 M NaCl in 50 mM NaOH at 30 µl/min. Biotinylated NP-fibrinogen was immobilised on flow cell 2 (1,500RU) and 3 (1500RU), whereas flow cell 1 was left without immobilised protein. NP-fibrin on SPR surface was generated as described previously for plasma fibrin[6]. 90 µl of 1U/ml thrombin in a buffer containing 10 mM HEPES, pH 7.4 and 140 mM NaCl, 2 mM $CaCl_2$, 0.05% tween 20 was injected at 2 µl/min over 45 min onto flow cell 3. The surface was regenerated with 90 µl 1 M NaCl for 3 min to remove bound thrombin. Dimeric GPVI was flowed over at concentrations up to 20 µM at a flow rate of 40 µl/min at 25 °C, by injecting 10 samples of the protein made from two-fold serial dilutions plus two buffer injections. Response signals obtained from flow cell 1 and buffer injections were subtracted in the data processing to obtain the final response curves. The data obtained from 5 lowest analyte concentrations where binding was observed were selected and fitted with 1:1 Langmuir model where k_a, k_d and K_D were obtained[7]. k_a, k_d and K_D values were presented as mean +/-SD; N=2. The highest analyte concentration curves were omitted in the fitting because they are less likely to follow the 1:1 interaction model than the lowest ones.

Supplemental Figures and Tables:

Fig I. Schematic representations of the structural composition of fibrinogen, fibrin (FXIII cross-linked) and their proteolysis products.
Fig II. Structure and SDS-PAGE profiles of recombinant GPVI-Fc (dimeric) and GPVI-ex (monomeric).
Fig III. Displacement of GPVI binding to immobilised fibrinogen in the presence of GPVI-Fc/GPVI monomer and binding of fibrinogen to immobilised GPVI-Fc and GPVI monomer.
Fig IV. SD test for GPVI-CRP-XL interaction using microscale thermophoresis.
Fig V. Stoichiometry analysis of GPVI-fibrinogen interaction using SPR.
Fig VI. GPVI-NP-fibrinogen interaction analysis using SPR with fitted K_D values.
Fig VII. Crystal structure of GPVI and its intermolecular interaction.
Fig VIII. SDS-PAGE profiles of recombinant WT/NP-fibrinogen.
Fig IX. Size exclusion chromatography overlay of GVPI monomer (his-tagged) and dimer.
Fig X. Proteolysis of fibrinogen and fibrin.
Fig XI. Mapping GPVI-fibrinogen binding sites using MST.
Fig XII. Mapping GPVI-fibrinogen binding sites using SPR (GPVI as analyte).
Fig XIII. Mapping GPVI-fibrinogen binding sites using SPR (GPVI as ligand).
Fig XIV. Measured and simulated SPR sensorgrams for monomeric GPVI and X-fragment interaction.

Fig XV. SDS-PAGE profiles of recombinant fibrinogen αC fragments.
Table I. Binding affinity of GPVI-ex, GPVI-Fc with fibrinogen and fragments measured by MST.
Table II. Binding affinity and kinetics parameters of GPVI-ex, GPVI-Fc binding to fibrinogen and fragments measured by SPR.

References

1. Onselaer M-B, Hardy AT, Wilson C, Sanchez X, Babar AK, Miller JL, Watson CN,Watson SK, Bonna A, Philippou H. Fibrin and D-dimer bind to monomeric GPVI. *Blood Adv.* 2017;1:1495-1504. doi:1182/bloodadvances.2017007732
2. Duval C, Profumo A, Aprile A, Salis A, Millo E, Damonte G, Gauer JS, Ariëns RA, Rocco MJJoT, Fibrinogen αC-regions are not directly involved in fibrin polymerization as evidenced by a "Double-Detroit" recombinant fibrinogen mutant and knobs-mimic peptides. *J Thromb Haemost.* 2020;18:802-81. doi: 10.1111/jth.14725
3. Duval C, Allan P, Connell SD, Ridger VC, Philippou H, Ariens RA. Roles of fibrin alpha- and gamma-chain specific cross-linking by FXIIIa in fibrin structure and function. *J Thromb Haemost.* 2014;111:842-850. doi: 10.1160/TH13-10-0855
4. Macrae FL, Duval C, Papareddy P, Baker SR, Yuldasheva N, Kearney KJ, McPherson HR, Asquith N, Konings J, Casini A et al. A fibrin biofilm covers blood clots and protects from microbial invasion. *J Clin Invest.* 2018;128:3356-3368. doi:10.1172/JCI98734
5. Smith KA, Adamson PJ, Pease RJ, Brown JM, Balmforth AJ, Cordell PA, Ariëns RA, Philippou H, Grant PJ. Interactions between factor xiii and the αc region of fibrinogen. *Blood.* 2011;117:3460-3468. doi: 10.1182/blood-2010-10-313601
6. Kearney KJ, Pechlivani N, King R, Tiede C, Phoenix F, Cheah R, Macrae FL, Simmons KJ, Manfield IW, Smith KA. Affimer proteins as a tool to modulate fibrinolysis, stabilize the blood clot, and reduce bleeding complications. *Blood.* 2019;133:1233-1244. doi: 10.1182/blood-2018-06-856195
7. Oshannessy DJ, Brighamburke M, Soneson KK, Hensley P, Brooks I. Determination of rate and equilibrium binding constants for macromolecular interactions using surface plasmon resonance: Use of nonlinear least squares analysis methods. *Anal Biochem.* 1993;212:457-468. doi: 10.1006/abio.1993.1355

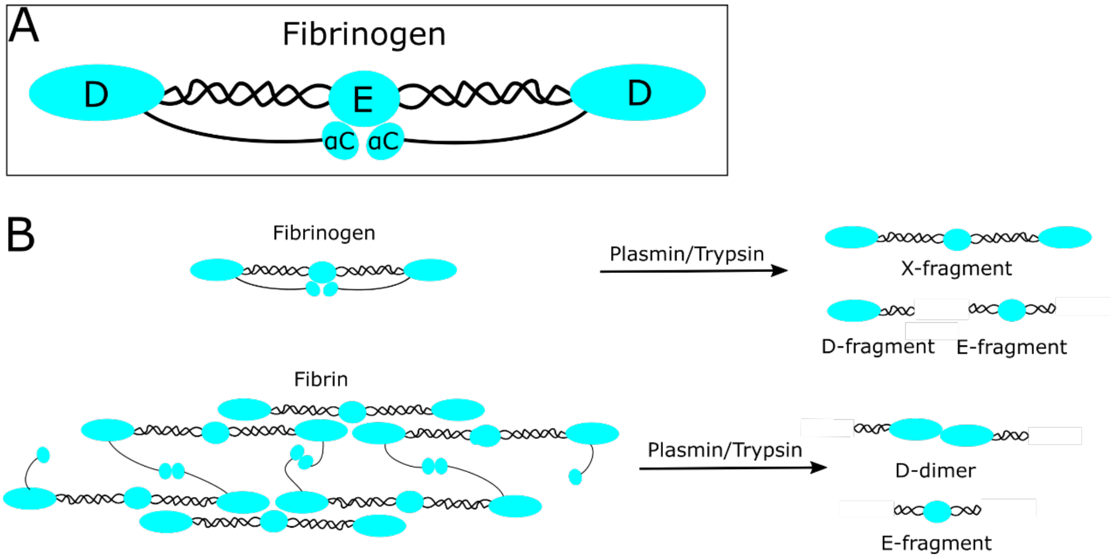

Fig I. Schematic representations of the structural composition of fibrinogen, fibrin (FXIII cross-linked) and their proteolysis products. (A) Fibrinogen is composed of several regions: 2 D-, 1 E- and 2 αC-regions. **(B)** Proteolytic digestion of fibrinogen by plasmin or trypsin generates X-fragment as an early product, and D-, E-fragments as final products. Similar proteolytic treatment to FXIII cross-linked fibrin gives D-dimer and E-fragment as final products.

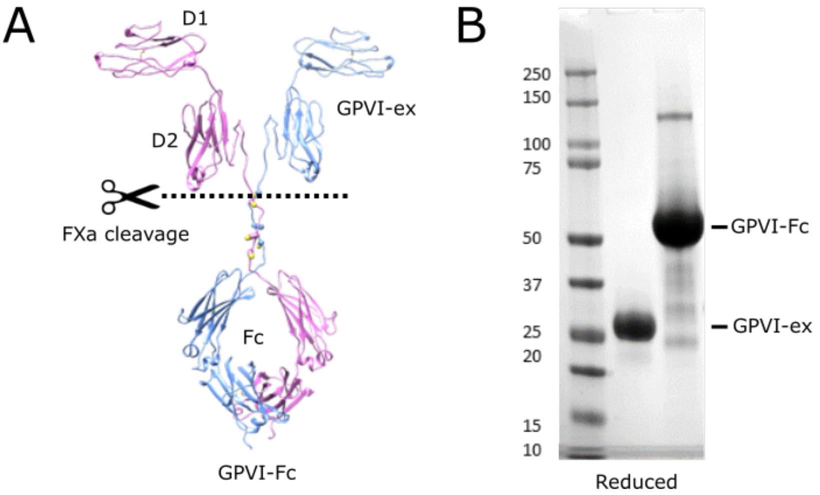

Fig II. Structure and SDS-PAGE profiles of recombinant GPVI-Fc (dimeric) and GPVI-ex (monomeric). (A) Predicted structure of GPVI-Fc using I-TASSER. The output structure with single peptide chain containing GPVI-ex and Fc from I-TASSER were superimposed with sialylated human IgG-Fc (PDBID: 4BYH) to generate the assembled dimeric GPVI-Fc structure. Three cystines were found between GPVI-ex and Fc of GPVI-Fc, likely holding the two chains by forming 3 disulphides. **(B)** SDS-PAGE of purified GPVI-Fc and GPVI-ex under reduced conditions.

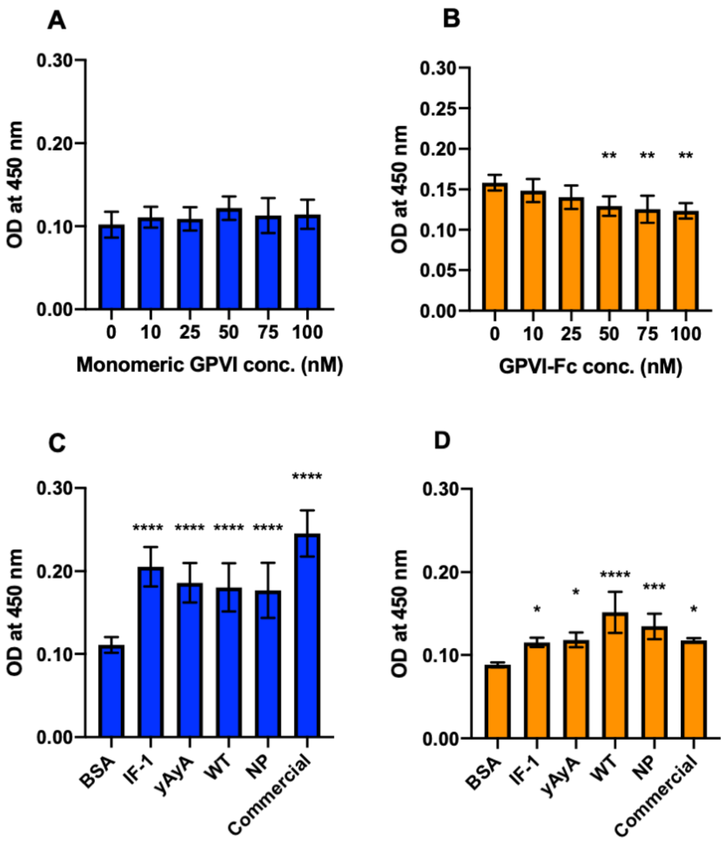

Fig III. Displacement of GPVI binding to immobilised fibrinogen in the presence of GPVI-Fc/GPVI monomer and binding of fibrinogen to immobilised GPVI-Fc and GPVI monomer. (A) Investigation of displacement of GPVI-Fc binding to immobilised IF-1 fibrinogen by increasing concentrations (0-100 nM) of GPVI monomer. **(B)** Displacement of GPVI monomer binding to immobilised IF-1 fibrinogen by increasing concentrations (0-100 nM) of GPVI-Fc. Binding of different types of purified fibrinogen (IF-1, γAγA, recombinant WT and recombinant NP) and un-purified commercially available fibrinogen (commercial) to immobilized GPVI-Fc **(C)** and GPVI monomer **(D)**. Differences in OD were compared between immobilised ligand and control; * $p \le 0.05$, ** $p \le 0.01$, *** $p \le 0.001$, **** $p \le 0.0001$.

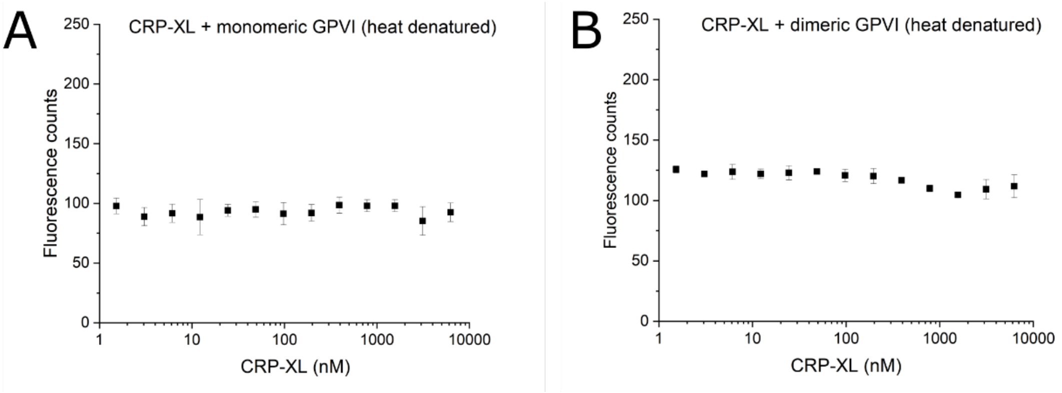

Fig IV. SD test for GPVI-CRP-XL interaction using microscale thermophoresis. The initial sample fluorescence intensity was re-measured for **(A),** FITC-GPVI-ex and CRP-XL and **(B),** FITC-GPVI-Fc and CRP-XL, at 75% LED power, after protein heat denaturation in the presence of SDS and DTT.

$$R_{max} = R_{ligand}\left[\frac{Mr_{analyte}}{Mr_{ligand}}\right]V_{ligand}$$

$$V_{ligand} = 1$$

$$R_{max(GPVI-Fc)} = R_{fibrinogen}\left[\frac{Mr_{GPVI-Fc}}{Mr_{fibrinogen}}\right] = 1500\frac{115\ kda}{340\ kda} = 507\ RU$$

$$R'_{max(GPVI-Fc)} = 803\ RU$$

Fig V. Stoichiometry analysis of GPVI-fibrinogen interaction using SPR. The theoretical maximum responses ($R_{max(GPVI\text{-}Fc)}$) assuming 1:1 interaction was calculated as 507 RU. The observed maximum response ($R'_{max(GPVI\text{-}Fc)}$) is 803 RU at 20 μM GPVI-Fc (the response unit at 85s of injection). The higher $R'_{max(GPVI\text{-}Fc)}$ than $R_{max(GPVI\text{-}Fc)}$ suggests non-1:1 interaction where more than one GPVI-Fc was involved in binding to one fibrinogen.

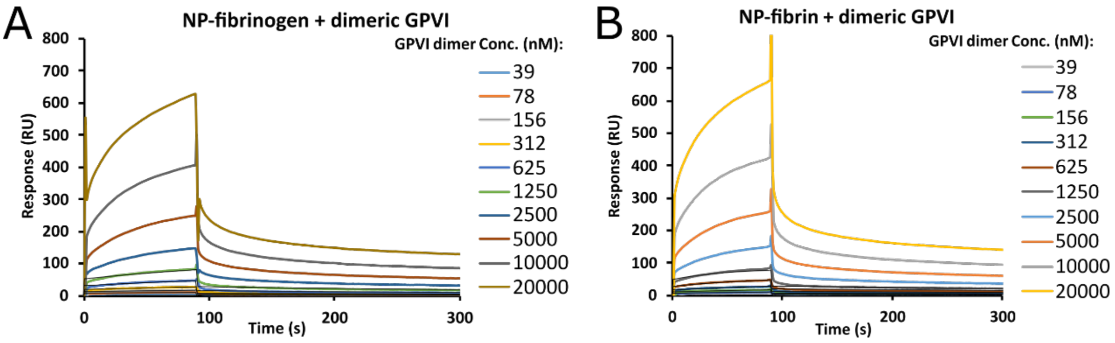

Fig VI. GPVI-NP-fibrinogen interaction analysis using SPR with fitted K_D values. (A) Dimeric GPVI binding to immobilised NP-fibrinogen at K_D of 127±57 nM. **(B)** Dimeric GPVI binding to immobilised NP-fibrin at K_D of 86±21 nM. Data shown is one

of two independent replicates from which K_D values were obtained through fitting individual data with Langmuir 1:1 interaction model. K_D values are presented as mean +/- SD; N=2.

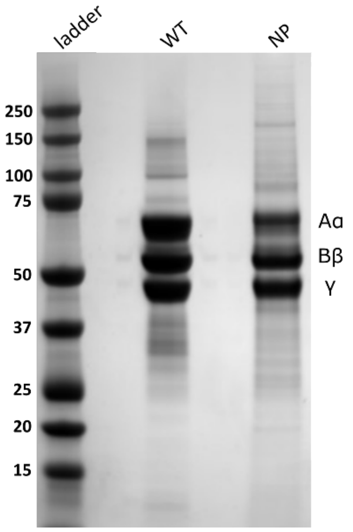

Fig VII. SDS-PAGE profiles of recombinant WT/NP-fibrinogen. Lane1: LMW marker; Lane2: WT-fibrinogen; Lane3: NP-fibrinogen.

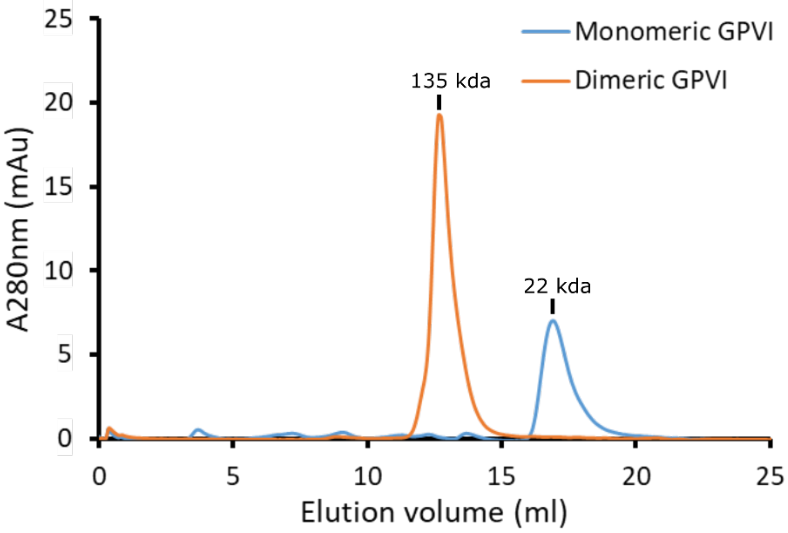

Fig VIII. Size exclusion chromatography overlay of GVPI monomer (his-tagged) and dimer. Both proteins eluted as single peaks. No formations of dimer or higher order oligomers were observed at this condition. Using a calibrated S200 column, the molecular weight of GPVI monomer and dimer were estimated at 22 and 135 kDa, respectively. These estimations are similar to their theoretical molecular weights calculated based on protein sequence (27 and 115 kDa for monomer and dimer, respectively).

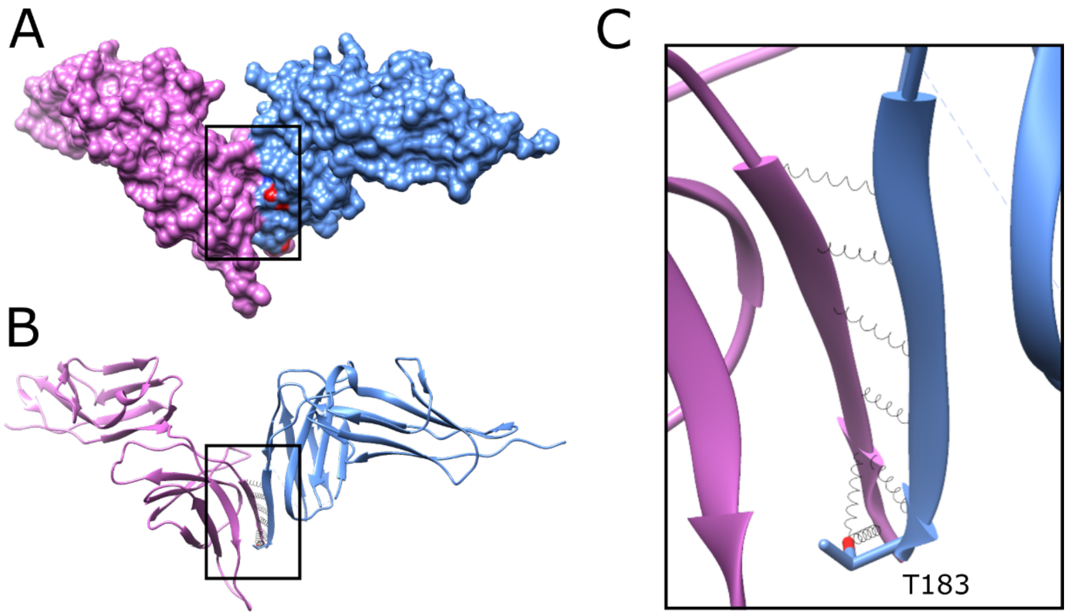

Fig IX. Crystal structure of GPVI and its intermolecular interaction. (A) Protein surface diagram showing the areas of possible GPVI intermolecular interaction. The two copies of GPVI were coloured in magenta and blue, respectively. **(B) and (C)** The 7 hydrogen bonds between the two molecules were indicated using black spring**.** 5 hydrogen bonds were formed through main chain-main chain interaction while 2 were formed by side chain-main chain interaction.

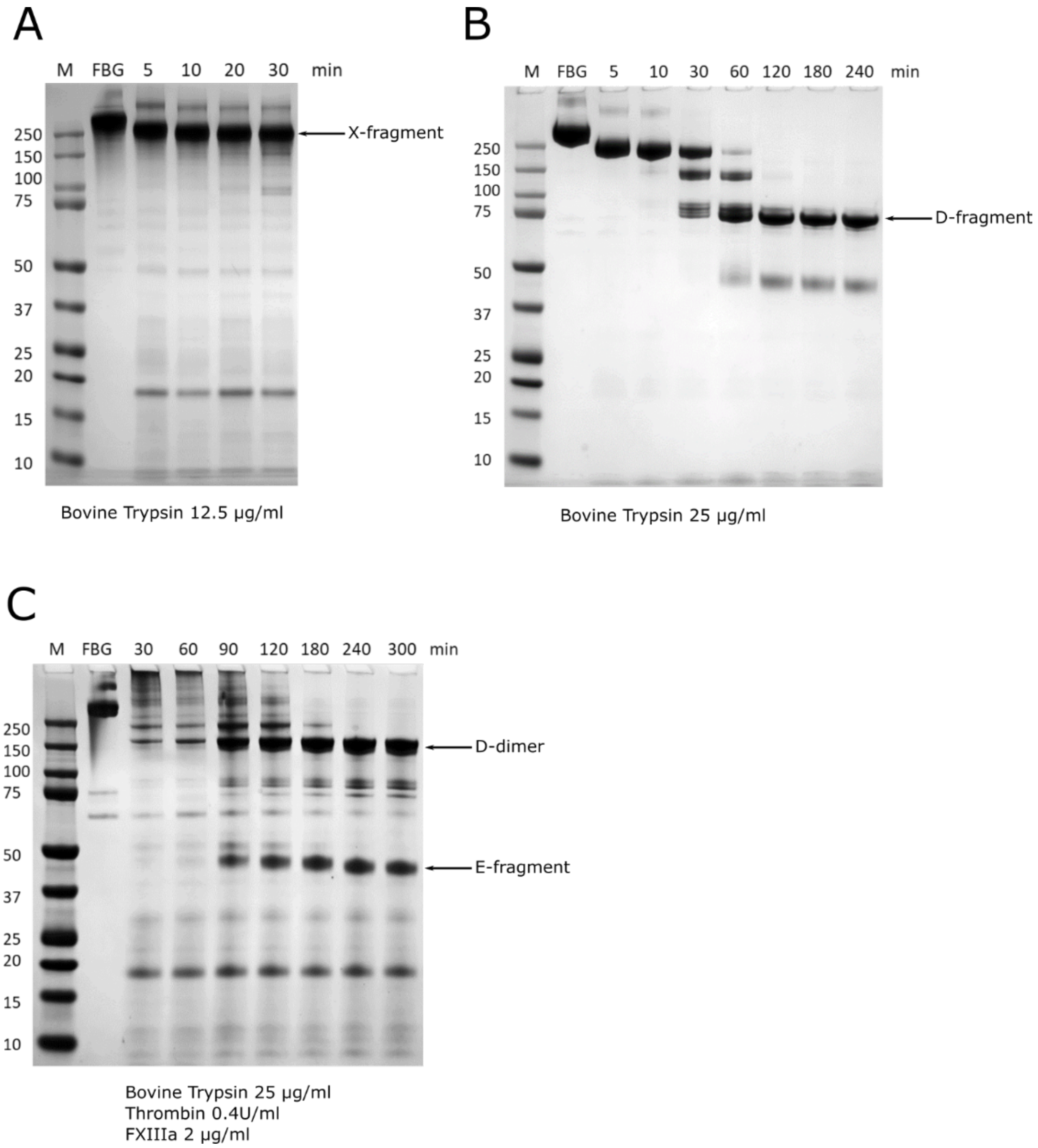

Fig X. Proteolysis of fibrinogen and fibrin. (A) Fibrinogen was proteolysed by bovine trypsin to produce X-fragment. **(B)** Longer incubation time of fibrinogen with bovine trypsin produced D-fragment. **(C)** FXIIIa cross-linked fibrin was digested by bovine trypsin to produce fibrin D-dimer and E-fragment.

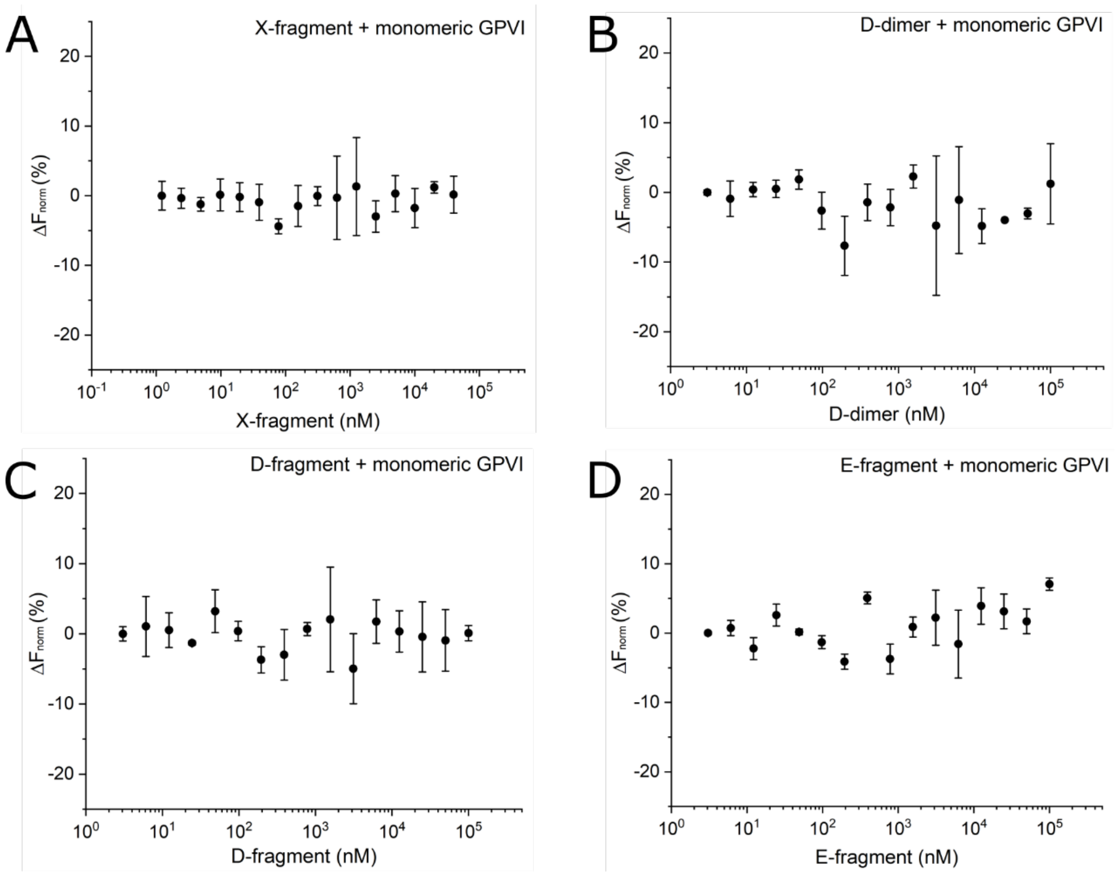

Fig XI. Mapping GPVI-fibrinogen binding sites using MST. Binding profiles of **(A)** X-fragment, **(B)** D-dimer, **(C)** D-fragment and **(D)** E-fragment to FITC-monomeric GPVI. No apparent binding was observed. N=3.

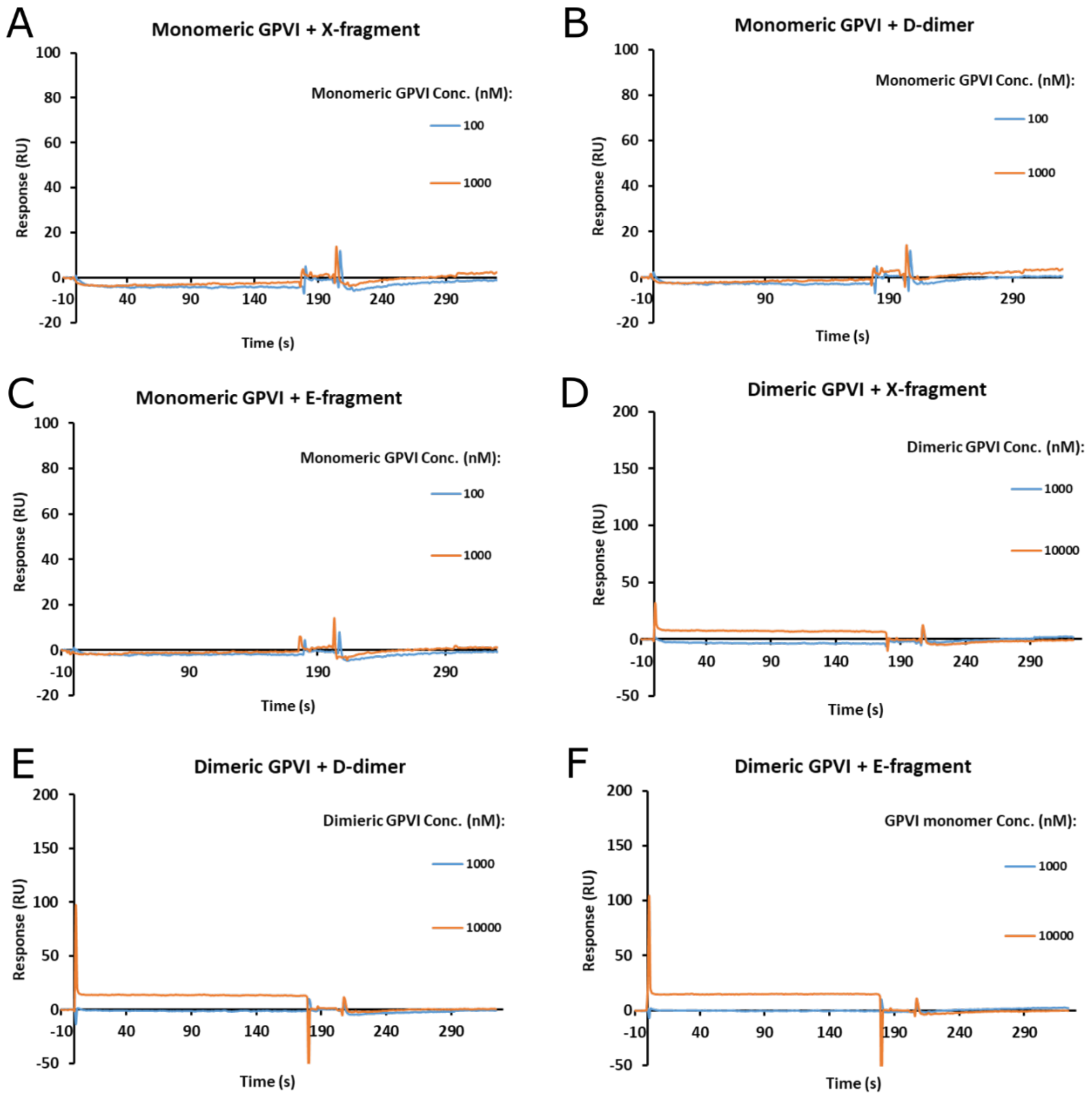

Fig XII. Mapping GPVI-fibrinogen binding sites using SPR (GPVI as analyte). Binding profiles of monomeric GPVI to immobilised **(A)** X-fragment, **(B)** D dimer and **(C)** E-fragment, respectively. No binding to the fragments was observed at the tested GPVI concentrations. Binding profiles of dimeric GPVI to immobilised **(D)** X-fragment, **(E)** D-dimer and **(F)** E-fragment, respectively. Binding was observed with rapid association and dissociation rate at 10 μM dimeric GPVI concentration. Similar binding profiles were also observed for fibrinogen fragments interaction with immobilised monomeric GPVI using SPR, with estimated affinity at >1mM based on simulation (See below Fig XIV).

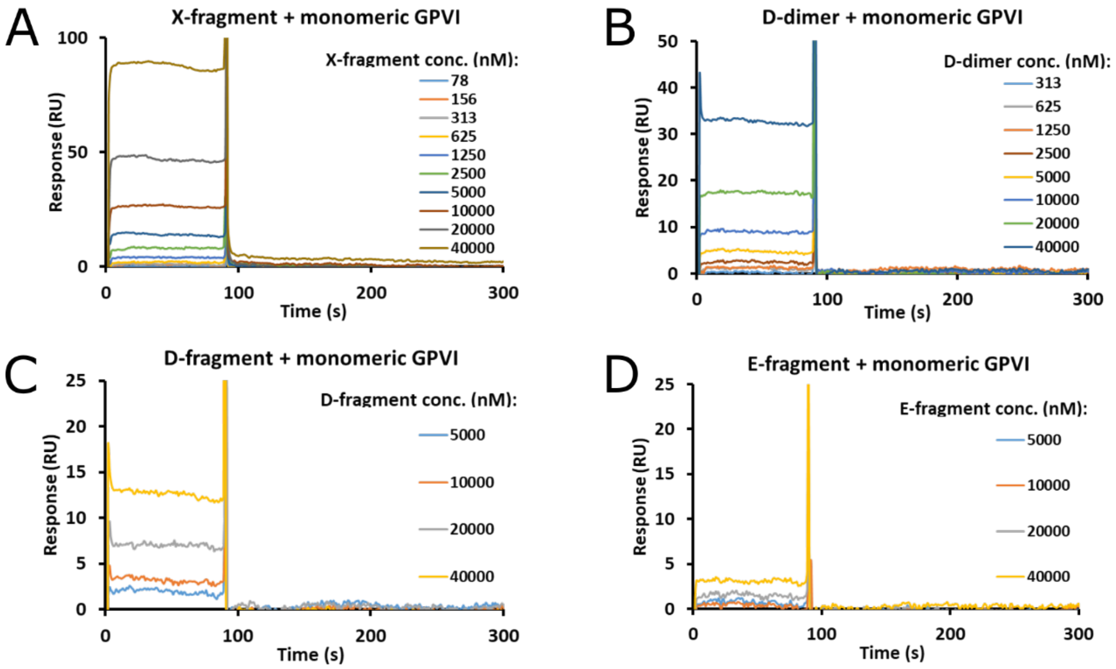

Fig XIII. Mapping GPVI-fibrinogen binding sites using SPR (GPVI as ligand). Binding profiles of **(A)** X-fragment, **(B)** D-dimer, **(C)** D-fragment and **(D)** E-fragment to immobilised monomeric GPVI. Due to the fast association and dissociation rates, kinetic analysis cannot be performed for monomeric GPVI and fibrin(ogen) fragments interaction. K_D was estimated using BIA simulation software at >1mM (See below Fig XIV).

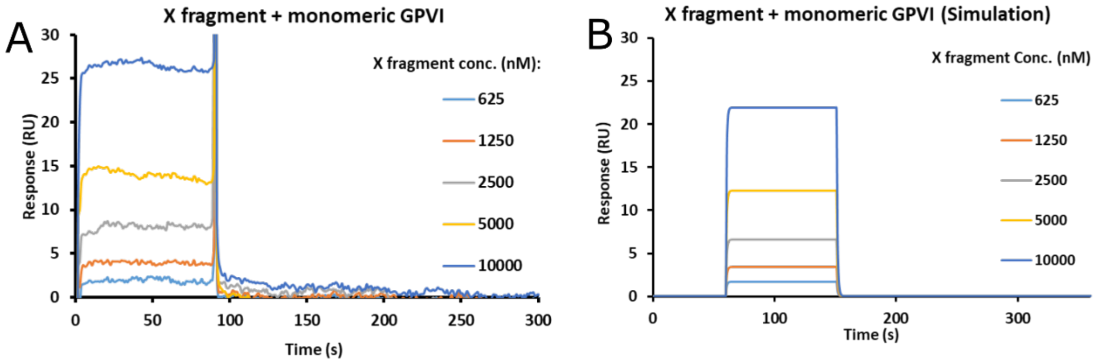

Fig XIV. Measured and simulated SPR sensorgrams for monomeric GPVI and X-fragment interaction. (A) X-fragment interacts with immobilised monomeric GPVI. **(B)** BIA simulation software simulated SPR sensorgrams using input association and dissociation rate constants for dimeric GPVI-X-fragment interaction. The association and dissociation rate constants were modified to render simulated sensorgrams similar to those observed in the experiment. The binding affinity was estimated at 128-fold lower than that determined for dimeric GPVI-X-fragment interaction, at >1mM.

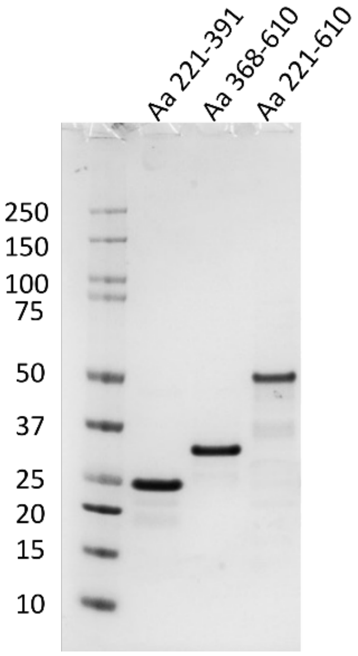

Fig XV. SDS-PAGE profiles of recombinant fibrinogen αC fragments. Lane1: LMW marker; Lane2: Aα221-391; Lane3: Aα368-610; Lane4: Aα221-610.

Table I. Binding affinity of GPVI-ex, GPVI-Fc with fibrinogen and fragments measured by MST.

	K_D (μM)	N
GPVI-ex + CRP-XL	5±0.8	3
GPVI-ex + Fibrinogen	>4	3
GPVI-ex + X-fragment	N.D.	3
GPVI-ex + D-dimer	N.D.	3
GPVI-ex + D-fragment	N.D.	3
GPVI-ex + E-fragment	N.D.	3
GPVI-Fc + CRP-XL	0.6±0.2	3
GPVI-Fc + Fibrinogen	0.1±0.002	3
GPVI-Fc + X-fragment	>2	3
GPVI-Fc + D-dimer	>20	3
GPVI-Fc + D-fragment	>20	3
GPVI-Fc + E-fragment	>20	2

Table II. Binding affinity and kinetics parameters of GPVI-ex, GPVI-Fc binding to fibrinogen and fragments measured by SPR.

	k_a ($M^{-1}S^{-1}$)	k_d (S^{-1})	K_D (μM)	χ^2	N
GPVI-ex + Fibrinogen	$5.7\pm0.2x10^2$	$2.2\pm0.05x10^{-3}$	2.4±0.3	3.3±2	3
GPVI-ex + X-fragment	-	-	>1000	-	3
GPVI-ex + D-dimer	-	-	>1000	-	3
GPVI-ex + D-fragment	-	-	>1000	-	3
GPVI-ex + E-fragment	-	-	>1000	-	3
GPVI-Fc + Fibrinogen	$8.5\pm0.2x10^3$	$6.1\pm0.1x10^{-4}$	0.046±0.005	57±19	3
GPVI-Fc + X-fragment	$9.9\pm1.4x10^3$	$5.4\pm0.7x10^{-2}$	9±1	4.9±2.1	3
GPVI-Fc + D-dimer	$1.5\pm0.02x10^3$	$3.9\pm0.4x10^{-2}$	28±3	1.8±0.2	3
GPVI-Fc + D-fragment	$1.3\pm0.4x10^3$	$3.3\pm1.4x10^{-2}$	26±5	0.3±0.1	3
GPVI-Fc + E-fragment	$4.1\pm2.6x10^2$	$1.9\pm0.7x10^{-2}$	51±15	0.2±0.05	2
GPVI-Fc + NP-fibrin	$1.7\pm0.01x10^4$	$1.4\pm0.3x10^{-3}$	0.086±0.02	2.2±0.8	2
GPVI-Fc + NP-fibrinogen	$1.4\pm0.2x10^4$	$1.8\pm0.6x10^{-3}$	0.13±0.06	5.1±0.9	2
GPVI-Fc + Aα 368-391	$6.5\pm0.8x10^3$	$4\pm1.1x10^{-3}$	0.6±0.09	0.9±0.1	2
GPVI-Fc + Aα 221-610	$1.7\pm0.1x10^3$	$3.9\pm0.5x10^{-3}$	2.3±0.2	3.8±2.5	2

Major Resources Table

In order to allow validation and replication of experiments, all essential research materials listed in the Methods should be included in the Major Resources Table below. Authors are encouraged to use public repositories for protocols, data, code, and other materials and provide persistent identifiers and/or links to repositories when available. Authors may add or delete rows as needed.

Animals (in vivo studies)

Species	Vendor or Source	Background Strain	Sex	Persistent ID / URL

Genetically Modified Animals

	Species	Vendor or Source	Background Strain	Other Information	Persistent ID / URL
**Parent - Male**					
**Parent - Female**					

Antibodies

Target antigen	Vendor or Source	Catalog #	Working concentration	Lot # (preferred but not required)	Persistent ID / URL
IgG-Fc	Thermo Fisher Scientific	62-8420	1:10,000	SH257188	Goat anti-Human IgG (Gamma chain) Cross-Adsorbed Secondary Antibody, HRP-1 mL
His-tag	Cambridge bioscience	A190-114P	1:10,000	A190-114P-19	Rabbit anti-6-His Tag Antibody HRP Conjugated

DNA/cDNA Clones

Clone Name	Sequence	Source / Repository	Persistent ID / URL
Fibrinogen Aα, Bβ and γ chain		Dr. Susan Lord (academic collaboration)	

Cultured Cells

Name	Vendor or Source	Sex (F, M, or unknown)	Persistent ID / URL
HEK-293T cells for the GPVI expression	Dr. Euan Baxter (academic collaboration)		
CHO cells for the fibrinogen expression	Dr. Susan Lord (academic collaboration)		

Data & Code Availability

Description	Source / Repository	Persistent ID / URL

Other

Description	Source / Repository	Persistent ID / URL
