# Supplementary figures and images for: GPVI (Glycoprotein VI) Interaction With Fibrinogen Is Mediated by Avidity and the Fibrinogen αC-Region
Source: Arterioscler Thromb Vasc Biol. 2021 Jan 21;41(3):1092–104. doi: 10.1161/ATVBAHA.120.315030 (PMC7901536; doi:10.1161/ATVBAHA.120.315030)

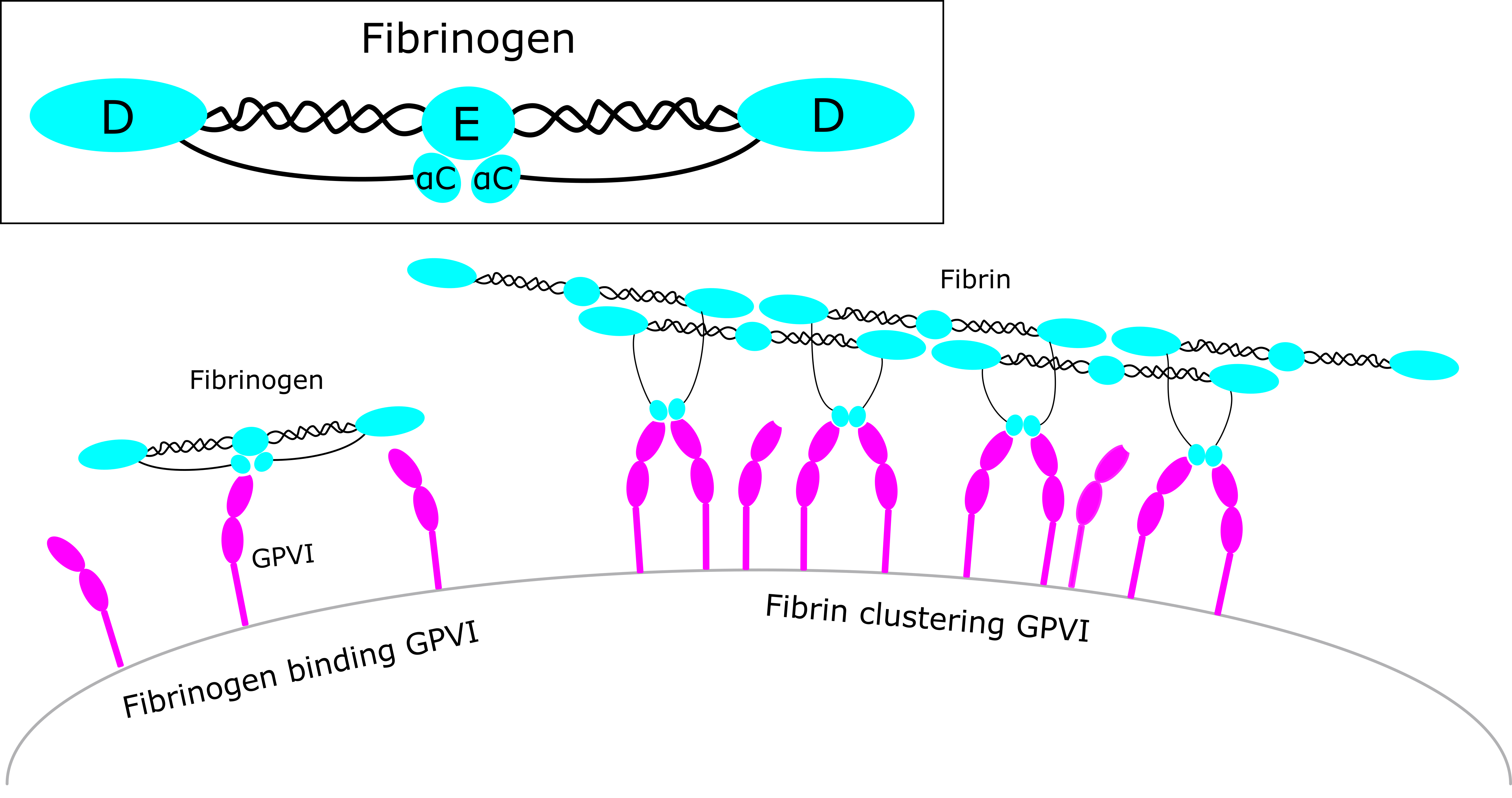

Supplement: Supplementary file 2 [file atv-41-1092-s002.jpg]
